# Definition and Test-Retest Reliability of a Monitoring Method Integrating Accelerometric Actigraphy and Bluetooth Indoor Location Tracking Applied in a Long-Term Residential Unit for Persons With Dementia: Longitudinal Observational Study

**DOI:** 10.2196/70188

**Published:** 2026-05-21

**Authors:** Marco Rabuffetti, Pietro Davide Trimarchi, Alessia Gallucci, Ilaria Carpinella, Elena Kisel, Maria Patrizia Andriani, Ennio De Giovannini, Gaia Bailo, Fabrizio Giunco, Maurizio Ferrarin

**Affiliations:** 1 IRCCS (Istituto di Ricovero e Cura a Carattere Scientifico) Fondazione Don Carlo Gnocchi Ente del Terzo Settore, via Capecelatro, 66, Milano, 20148, Italy, 39 3382509295, 39 0239210325; 2 Centro Medico Riabilita Cooperativa Sociale Mano Amica Onlus, Schio, Italy

**Keywords:** dementia, long-term residency unit, physical activity, location tracking, wearable sensor, social actigraphy

## Abstract

**Background:**

Dementia has an impact on the physical activities performed daily in a social context. Sleeping and resting, in general, are also affected by dementia. Monitoring techniques based on miniaturized wearable sensors and on sensorized environments allow for actigraphic recordings and location tracking. The availability of contemporaneous physical activities profile led to quantify, in the social actigraphy approach, the level of correlation between individuals living in the same environment.

**Objective:**

This study has two main objectives: (1) to define a methodology for actigraphic recordings, based on wearable accelerometers, and on location tracking, based on Bluetooth wearable technology, and to apply it in a well-defined social context, a long-term care residential unit for people with dementia; and (2) to quantify test-retest reliability of the indices obtained by the monitoring methodology.

**Methods:**

Persons with dementia living in the long-term care unit have been equipped with miniaturized wearable sensors, an accelerometer at their dominant wrist, and a Bluetooth beacon at their ankle for 7 days. The raw recordings allowed for computing indices related to physical activity intensities, to the occurrence of walking bouts, to the efficiency of sleep and waking phases, to social interactions between individuals, and to locations preferably occupied. The 7-day session was repeated at short (3 weeks) and long (3 months) terms in order to quantify the test-retest reliability of the indices.

**Results:**

Twenty-five persons with dementia were enrolled, 4 of them dropped out, and valid data were obtained, in the different sessions, from 19 to 21 individuals of the recruited group. Control data from 10 age-matched healthy participants were derived from published datasets. As a group, compared with age-matched healthy participants, persons with dementia showed a comparable duration of phases of no activity and of light activity (energy cost lower than 3 metabolic equivalents of tasks [METs]), a relevantly lower duration (−84.3%) of phases of moderate activity (energy cost ranging from 3 to 6 METs), and substantial absence (−100%) of phases of vigorous activity (larger than 6 METs); moreover, daytime and nighttime were characterized by comparable wake and sleep, respectively, efficiency; finally, as to the social interactions, persons with dementia showed a lower correlation of their motor activity profiles (−53.1%). The test-retest reliability was excellent for physical activity indices (intraclass correlation coefficients ranging from 0.76 to 0.98), good for social indices (0.65‐0.67), excellent for sleep or wake efficiency (0.74‐0.89), and fair for location tracking indices (0.37‐0.78).

**Conclusions:**

The considered methodology, particularly concerning accelerometry, proved to be feasible, informative, and with a good to excellent test-retest reliability. Interestingly, the methodology clearly identified behaviors, such as wandering, in a minority of individuals inside this study’s group of persons with dementia, thus supporting a possible clinical use of the methodology.

## Introduction

The number of people with dementia is estimated to increase from 57.4 million cases worldwide in 2019 to 152.8 million cases in 2050, with the female-to-male ratio of 1.69 in 2019 being substantially stable and the projected increases in cases largely attributed to population growth and population aging [1].

One of the most relevant aspects in the management of individuals with dementia, even in mild-to-moderate stages, is related to movement [2]. The transition from a life without illness to a life dealing with alterations in spatial-temporal orientation or critical judgment and perception of dangers requires a delicate balance between the freedom the person still demands (moving around the house, walking, or going out) and the dangers they are exposed to, such as the risk of falls, disorientation, and domestic and road accidents. For these reasons, both in domestic and residential settings, there is a significant use of physical or pharmacological restraint, which sometimes may also be improper or untimely [3-5]. Alternatively, or as an integration to such restraints, there are environmental and behavioral management strategies, involving architectural or environmental adaptations that reduce risks and dangers, and/or facilitation, validation, and orientation support strategies [6]. Accordingly, special Alzheimer Units (“Nuclei Alzheimer”), long-term residential wards specific for persons with dementia, introduced by the Italian region Lombardy legislation in the mid nineties [7], are specifically designed and organized to promote free movement and activity in a safe and supervised environment, and where specific interventions may be carried out with safety and efficiency.

In order to support supervision of motor behaviors [8], particularly in nursing settings, easily manageable methodologies can help the caregivers. Current technologies based on wearable accelerometers support activity tracking [9,10]. Interestingly, these approaches may support group studies which provide information about the interplay among persons, thus implementing the so-defined social actigraphy [11]. Additionally, systems based on wearable Bluetooth beacons and antennas [12], similar to the contact tracing methods adopted during the COVID-19 pandemic [13], can support indoor location tracking in order to identify the room or environment the participant is currently in [14-16]. Such possibilities of using continuous measures of the quantity and quality of movement and tracking of location represent a relevant contribution to assessments based on tests, surveys, and questionnaires [17]. The integration of an activity tracking system and a location tracking system has already been considered in order to compute activity-related indices specific to indoor location. This approach proved to be able to better distinguish between at-risk patients who can gain independence vs the patients who are rehospitalized [18], and, when applied to a longitudinal study, provided information concerning patients receiving rehabilitation at a nursing facility [19].

When considering instrumented monitoring of activity and location, it is important to underline the inherent variability associated with free behavior, which requires us to monitor human behavior for a relevant time duration, starting from a single day [20], the basic circadian cycle, up to 1 week [11] and more [21], to obtain reliable and valid outcome indices converging to stable values [22,23]. Still, outcome indices show interindividual and intraindividual variability that would require proper experiments to be characterized in terms of test-retest reliability. Such variability thresholds allow for the identification of only significant deviations that may be predictive of critical situations, such as abnormal movement patterns or deviations from usual behaviors, suggestive of a changed, eventually worsened, condition. Intraindividual test-retest variability has already been considered for physical activity questionnaires on persons with dementia [24-26] and for actigraphic outcome indices related to movement [27-29].

The objective of the present study is to define a composite method for actigraphic monitoring [11,30] and indoor location tracking in a community residence and to perform an experimental campaign in an Alzheimer Unit, a long-term residential care facility for persons with dementia, to quantify the test-retest reliability of the resulting indices.

## Methods

### Ethical Considerations

This study and related documents were approved by the local Ethics Committee (Comitato Etico IRCCS [Istituto di Ricovero e Cura a Carattere Scientifico] Regione Lombardia, Sezione IRCCS Fondazione Don Carlo Gnocchi, ID 07_07/04/2021, April 7, 2021, amended on May 26, 2023). Due to severe dementia, informed consents were obtained from legal tutors of the participants. Any data or information was recorded in an anonymized form. No compensation was provided to participants.

### Participants

Participants were recruited from the Alzheimer Unit of the Palazzolo Institute, Don Carlo Gnocchi Foundation, Milan. As it is a pilot study, no minimum sample size has been identified, but all resident guests of the experimental site were contacted for possible enrollment.

The inclusion criteria were an autonomous locomotor capacity (even with the help of walking aids such as, for example, a cane or a crutch) and the frequentation of common areas of the residential unit. The exclusion criteria were the presence of behavioral disorders incompatible with the research objectives (delirium, psychomotor agitation requiring psychoactive pharmacological interventions, acute clinical events limiting autonomous movement, and nonneurological pathologies strongly limiting motor activities), the need for assistance for walking by a caregiver, any dermatological problems preventing contact with wearable sensors, any confined conditions or quarantine for COVID-19 risk management.

Reference data from age-matched healthy participants were extracted from previously published datasets [11,30] in order to build a control group with a comparable average age.

### Clinical and Functional Assessment

The chosen assessment battery included Hendrich II Fall Risk Model [31], Tinetti Scale [32] when applicable, Barthel Index [33], Mini-Mental State Examination [34], Clinical Dementia Rating [35], and Scheda di Osservazione Intermedia di Assistenza [36]. Moreover, an Event Monitoring Diary (clinical events, changes in pharmacological and nonpharmacological interventions, falls, discomfort, and/or incongruous interaction with sensors, and temporarily leaving the ward) was compiled during the experiment.

### Actigraphic Monitoring

**Figure 1.**
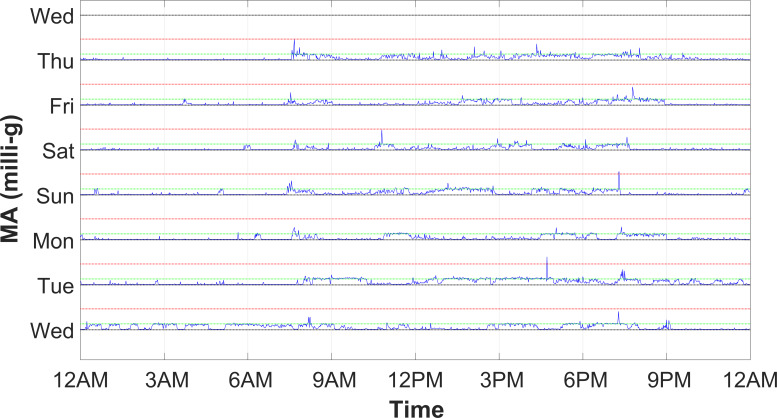
Seven-day profile of the MA index of a guest of the long-term care residential unit. Y-grid dotted lines in green and red mark, respectively, values 100 (transition value from LPA to MPA) and 350 (transition value from MPA to VPA) milli-g above black dotted baselines. LPA: light physical activity; MA: motor activity; milli-g: measurement unit is one thousandth of Earth gravity constant g; MPA: moderate physical activity; VPA: vigorous physical activity.

Participants wore, at their dominant wrist, a wristwatch-like waterproof device including a triaxial accelerometer for 1 week. Accelerometric raw data (measurement unit is one thousandth of earth gravity constant g, termed in the following as milli-g), sampled at 50 Hz, were stored in a resident memory (Geneactiv, Activinsights). The device was not to be removed unless the participant was willing to. The 3 acceleration components allowed for the computation of the vector norm, from which the motor activity (MA) index was derived as the SD of the vector norm in 1-minute epochs [37], thus resulting in a 7-day MA profile composed of 1440 values per day (Figure 1).

**Figure 2.**
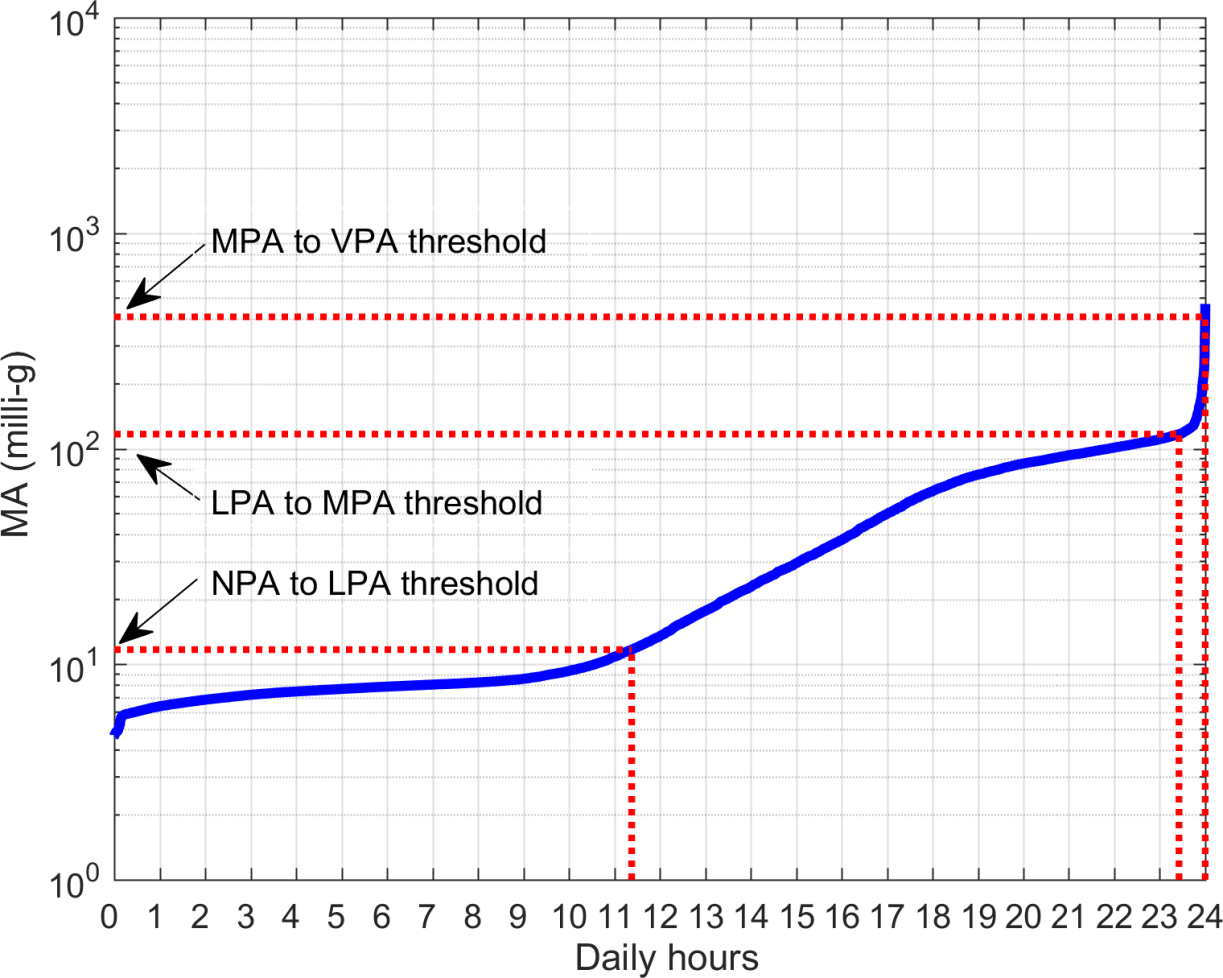
The distribution of the MA values measured for 7 days is presented in Figure 1. The predefined MA thresholds (identified by values on the y-axis) allow for quantifying the duration (as an interval on the x-axis) on a daily basis of the phases of NPA, LPA, MPA, and VPA (this latter one is almost absent in the shown example as well as in the whole dataset). LPA: light physical activity; MA: motor activity; MPA: moderate physical activity; NPA: no physical activity; VPA: vigorous physical activity.

A sorting operation on the MA profile produced a distribution plot (Figure 2) which, through proper thresholds (10 milli-g for transition from no to light physical activity [LPA], 100 milli-g for light to moderate, and 350 milli-g for moderate to vigorous, for a sensor located on the nondominant wrist) [38], eventually corrected for dominant side (previous threshold values multiplied by Coeff_Dominant_=1.16926), allowed computation of physical activity indices: the percentage duration of the phases with no physical activity (NPA), LPA, moderate physical activity (MPA), and vigorous physical activity (VPA).

An additional analysis was performed on the raw acceleration profile to detect the occurrence of regular patterns that are likely to be associated with a pseudoperiodic function such as locomotion. The raw signal was windowed in 4-second epochs, and an autocorrelation analysis was applied to the signal window. The possible occurrence of lagged peaks of the autocorrelation function was searched, adopting a threshold of 0.7 (a perfect periodic phenomenon has a unitary peak value, while white noise is denoted by null values of the autocorrelation function), which had been set, based on trial-and-error attempts [39]. The duration of such pseudoperiodic behavior (PPB) phases was quantified by their percentage duration (PPB).

Two more indices were computed to quantify the quality of nighttime sleep and daytime wake time: nighttime sleep efficiency (SEff), the percentage duration of NPA phases between 11 PM and 7 AM, the a priori “night” time interval, which is related to SEff [40]; analogously, daytime wake efficiency (WEff), the percentage duration of LPA, MPA, or VPA (ie, the complementary duration of no activity phases) between 9 AM and 7 PM, the a priori “day” time interval, which quantifies WEff. The choice of the strict timing for “day” and “night” time intervals was based on the daily routines established within the long-term care unit: all guests are expected to be in bed during the “night” interval and awake during the “day” interval, while this assumption is not true for transition intervals (7 AM-9 AM and 7 PM-11 PM).

### Location Tracking

**Figure 3.**
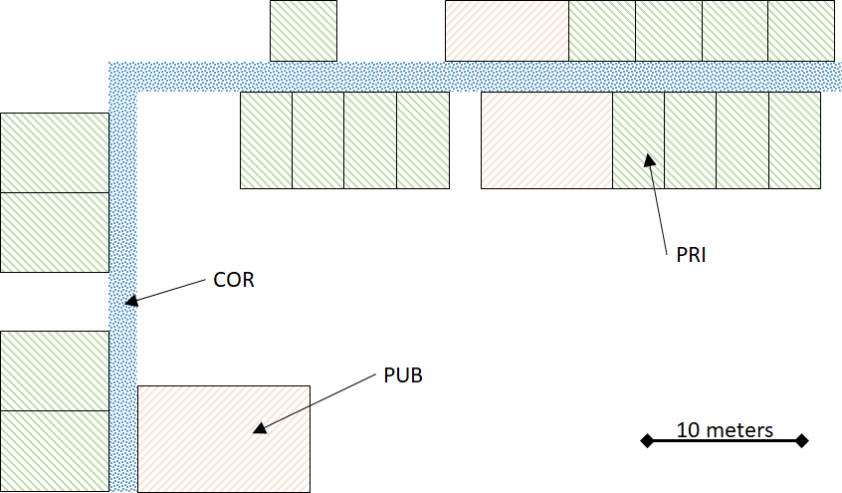
Schematic map of the long-term care residential unit where the monitoring experiments took place. Different colors or patterns allow for identification of PRIs, PUBs, where social daily activities take place, and corridors. COR: corridor; PRI: private room; PUB: public room.

The chosen location tracking system is composed of individual wearable devices and a network of environment fixed-position antennas (TapMyLife Inc). Monitored participants wore a miniaturized Bluetooth beacon (dimensions 32 mm × 23 mm × 3.3 mm, weight 4.0 grams) at one of their ankles by means of a biocompatible plastic bracelet. Such a device continuously emits a unique ID code, possibly received by a Bluetooth receiving antenna positioned nearby (according to the Bluetooth technology, approximately up to 10 meters in open space). All environments where participants resided (private bedrooms and any common spaces they spend time in, such as dining rooms, living rooms, corridors, etc) were equipped with Bluetooth receiving antennas: 1 antenna for each private room (PRI; 13 double rooms and 4 single rooms), 2 antennas for each large public room (3 PUBs), and 7 antennas along the main corridor, as shown in Figure 3. The frequency of the Bluetooth emitter is partly determined by the asynchronous Bluetooth protocol, generally in the magnitude order of 1 Hz. As a single Bluetooth emission can be received by several antennas, a proprietary signal algorithm identified the closest antenna and updated the beacon location (ie, the identified receiving antenna position) only when the beacon switched to a new antenna. Therefore, the source raw data provided by the location tracking system was natively asynchronous, and the current location of a beacon was determined by the position of the last antenna that detected the beacon itself. In order to summarize the location data, the percentage values of time spent in PRIs, common living rooms (PUBs), and corridors were computed.

### Experimental Sessions

The single monitoring experiment lasted for 7 days. Three experimental sessions were performed in July (Session A), September (Session B), and October (Session C) 2023. For short-term repeatability assessment (3-week time interval), Sessions B and C were compared. For long-term repeatability assessment (3-month time interval), the Sessions A and C were considered.

### Outcome Variables

The variables computed starting from the actigraphic recordings were:

- NPA, LPA, MPA, and VPA: the percentage duration of NPA (1 metabolic equivalent of task [MET], MA≤10), LPA (1‐3 MET, 10<MA≤100), MPA (3‐6 MET, 100<MA≤350), and VPA (>6 MET, MA>350) [38], where MET stands for metabolic equivalent of task, a standardized unit of measurement for the energy cost of physical activity, being 1 MET equal to the metabolic energy expended while sitting at rest;
- PPB: the percentage duration of pseudoperiodic behavior, whose occurrence was identified in 4-second epochs of the acceleration norm in which the autocorrelation coefficient was larger than 0.7 [39];
- CC: the cross-correlation coefficient between contemporaneous physical activity MA profiles in couples of participants [11], its unit is a real value from 0 (absence of cross-correlated physical activities) to 1 (perfectly synchronous physical activity);
- SEff: nighttime sleep efficiency, the percentage of time between 11 PM and 7 AM in which MA was classified as NPA [40];
- WEff: daytime wake efficiency, the percentage of time between 9 AM and 7 PM in which MA was classified as LPA, MPA, or VPA;
- PRI, PUB, and corridor: the percentage duration of time spent in PRIs, that is, bedrooms (PRI), in PUBs, that is, social spaces where guests of the long-term care residential unit had their meals and performed leisure activities (PUB), and in public spaces, that is, main corridors.

Indices were computed on the 7-day-long recording. When the full-length recordings included a portion of scratch recording for any reason, for example, because of participants taking off the sensor, the scratch portion was removed from the recording, and the remaining time duration was further reduced to the highest possible multiple of 24 hours. The previous algorithm allowed for the computation of the indices on recordings whose length was a multiple of the basic 24-hour circadian cycle.

### Statistical Analyses

The difference between outcome variables as obtained from control data and from pooled experimental data was quantified as percentage variation of median values and presented as boxplots. The difference was assessed by the Wilcoxon rank sum test (as implemented by “ranksum” function in MATLAB software, version R2016a, The MathWorks Inc), a nonparametric test for 2 independent populations.

Test-retest reliability was presented as scatterplots and assessed through the intraclass correlation coefficient (ICC). ICC was computed with a 95% CI, using a 2-way mixed-effect, absolute agreement, single measurement model. Significance level was set at 0.05. The significance level was corrected for multiple comparisons according to Bonferroni-Holm.

The adopted guidelines for interpreting ICC [41] meant “poor” for values less than 0.40, “fair” for values between 0.40 and 0.59, “good” for values between 0.60 and 0.74, and “excellent” for values between 0.75 and 1.00.

## Results

The setup of technical infrastructure (BLE antennas) took place at the beginning of 2023. The data collection phase lasted from June to October 2023. The 30 guests of the long-term care residential unit were considered for this study. It was not obtained or was not possible to obtain informed consent from 5 persons, who were therefore not recruited for this study. A total of 25 guests of the long-term care residential unit provided informed consent, through their legal tutors, to participate in this study. Their demographic data and clinical scores are summarized in Table 1. Noticeably, some clinical tests were not administered (marked as “n.adm.” in the table) to some persons because of their inability to receive them. At the time of the first experiment, 4 participants denied their participation by refusing to be equipped with the sensors. Therefore, the experimental group included 21 participants (15 women and 6 males); their age was 83.4 years on average, SD 10.1 years, ranging from 69 to 97 years plus 1 younger outlier aged 55 years. Four participants rejected the wrist sensor after a variable duration from 2 days to 6 days (but the collected data lasted more than 24 hours, thus they were allowed to be included in the analysis). At the time of the second experiment, 2 more participants denied their participation and dropped out, and a third one rejected the wrist sensor after 2 days of data collection. At the time of the third experiment, 2 participants rejected the wrist sensor after 1 or 2 days, and 1 participant removed the wrist sensor, and it was lost. Therefore, the dataset for the analysis included recordings from 21 participants for the location tracking, while the actigraphic assessment included 21 participants in the first experiment, 19 participants in the second experiment, and 18 participants in the third experiment. All the observed sensors’ rejections were clearly expressed by the participants with signs of discomfort about that “alien” presence at their wrist or ankle. Though it was not possible to clarify with them the reason why, while possible explanations, such as a tight strap or bracelet, were excluded by the experimenter’s supervision. Therefore, the rejections were considered a behavioral aspect of the dementia condition.

**Table 1.** Demographic data and clinical scores of this study’s participants.

Subject ID	Sex (F^a^/M^b^)	Age (y)	CDR^c^ [35]	MMSE^d^ [34]	Barthel [33]	SOSIA^e^ [36]	Tinetti et al [32]	Hendrich II Fall Risk [31]
S01^f^	F	84	3	3	44	7	22	6
S02	M	69	3	N.adm.^g^	32	5	22	7
S03^f^	F	79	3	N.adm.	55	7	26	6
S04	F	89	3	16	57	5	21	6
S05	M	88	3	N.adm.	59	7	21	7
S06	F	85	3	N.adm.	34	7	23	5
S07	F	91	3	N.adm.	37	5	21	5
S08	F	82	3	N.adm.	40	7	23	6
S09	F	80	3	N.adm.	33	5	23	5
S10	F	97	3	9.4	73	7	23	5
S11	F	87	3	11.2	60	7	21	6
S12	F	79	3	N.adm.	26	5	N.adm.	5
S13	M	87	3	N.adm.	29	5	N.adm.	7
S14	F	88	3	7	61	7	21	6
S15	F	94	2	13	59	7	24	6
S16^f^	M	63	3	N.adm.	44	5	22	7
S17^f^	F	90	3	N.adm.	56	5	22	5
S18	M	69	3	N.adm.	20	5	N.adm.	6
S19	F	84	3	7	68	7	23	6
S20	M	75	3	N.adm.	26	5	20	7
S21	F	85	3	16	61	5	22	6
S22	F	92	3	N.adm.	34	7	20	7
S23	F	55	3	N.adm.	31	5	19	6
S24	F	96	3	N.adm.	26	5	N.adm.	8
S25	M	80	3	3	34	5	23	7

a F: female.

b M: male.

c CDR: Clinical Dementia Rating.

d MMSE: Mini Mental State Examination.

e SOSIA: Scheda di Osservazione Intermedia di Assistenza.

f Indicates participants dropped out before Session A experiment.

g N.adm.: not administered.

No major health episode possibly influencing mobility was observed (and recorded in the Event Monitoring Diary) among the participants during the 3-month span in which experiments took place.

The control group included 10 healthy persons living in their own homes, 5 males and 5 females, aged 82.1 years on average (SD 2.1 years), ranging from 80 to 87 years, and were extracted from the dataset of a previously published study [11,30]. For the interaction outcomes (CC index), reference data from 40 healthy individuals (20 females and 20 males), whose mean age was 72 years, SD 7.6 years, ranging from 58 to 87 years) from another study [11] were considered.

The summary of all results is presented in Table 2.

**Table 2.** Summary statistics of resulting indices.

Index	Controls median (IQR)	Cases session A median (IQR)	Cases session B median (IQR)	Cases session C median (IQR)	Wilcoxon raw *P* value (after Holm correction)	Short-term ICC^a^ median (95% range)	Long-term ICC median (95% range)
NPA^b^	37.0 (7.0)	50.4 (21.9)	44.1 (16.9)	48.8 (17.7)	.01 (n.s.^c^)	0.92 (0.81‐0.97)	0.88 (0.71‐0.95)
LPA^d^	37.9 (4.1)	38.6 (8.8)	41.1 (17.7)	40.2 (16.4)	.20 (n.s.)	0.86 (0.66‐0.95)	0.76 (0.45‐0.90)
MPA^e^	23.3 (8.4)	2.7 (8.0)	3.7 (0.3)	3.9 (11.9)	<.001 (<.05)	0.96 (0.90‐0.98)	0.84 (0.62‐0.94)
VPA^f^	0.2 (0.5)	0.0 (0.0)	0.0 (0.0)	0.0 (0.0)	<.001 (<.05)	N.c.^g^	N.c.
PPB^h^	0.8 (1.6)	0.3 (3.2)	0.7 (5.9)	1.0 (4.1)	.68 (n.s.)	0.83 (0.59‐0.93)	0.95 (0.87‐0.98)
CC^i^	0.28 (0.08)	0.14 (0.13)	0.14 (0.17)	0.13 (0.11)	<.001 (<.05)	0.67 (0.58‐0.75)	0.65 (0.55‐0.73)
SEff^j^	82.8 (4.7)	75.0 (24.6)	74.7 (29.2)	77.1 (25.4)	.09 (n.s.)	0.89 (0.73‐0.96)	0.78 (0.50‐0.91)
WEff^k^	88.9 (10.7)	70.3 (28.1)	78.3 (22.1)	75.0 (15.3)	.03 (n.s.)	0.74 (0.42‐0.89)	0.81 (0.57‐0.93)
PRI^l^	N/A^m^	59.5 (5.2)	52.9 (10.0)	56.0 (6.7)	N/A	0.37 (0.07‐0.68)	0.48 (0.06‐0.75)
PUB^n^	N/A	35.3 (7.5)	29.6 (10.9)	29.8 (14.0)	N/A	0.78 (0.54‐0.91)	0.48 (0.06‐076)
COR^o^	N/A	5.3 (12.1)	16.7 (15.3)	17.0 (19.9)	N/A	0.59 (0.22‐0.81)	0.47 (0.05‐0.75)

a ICC: intraclass correlation coefficient.

b NPA: no physical activity.

c n.s.: not significant.

d LPA: light physical activity.

e MPA: moderate physical activity.

f VPA: vigorous physical activity.

g N.c.: not computed.

h PPB: pseudoperiodic behavior.

i CC: cross-correlation coefficient.

j SEff: sleep efficiency.

k WEff: wake efficiency.

l PRI: private room.

m N/A: not available.

n PUB: public room.

o COR: corridor.

The analytical presentation of selected indices’ values is provided in the form of a boxplot, which also displays any potential outliers. The ICC values are interpreted as “excellent” when larger than 0.75, “good” when between 0.60 and 0.74, “fair” when between 0.40 and 0.59, and “poor” otherwise.

**Figure 4.**
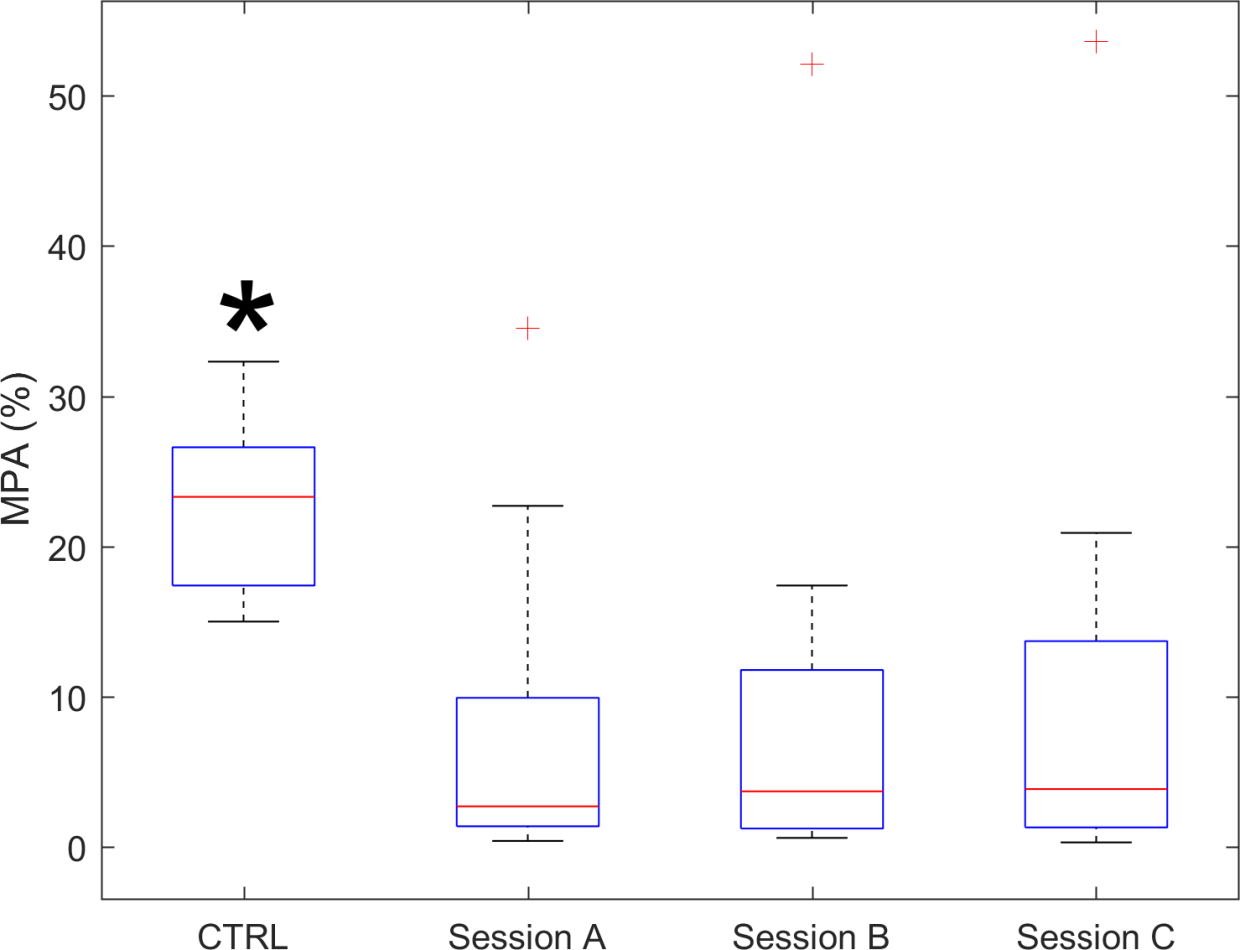
MPA index for CTRL and for the 3 sessions of the experimental group. Red “+” marks indicate outlier individuals. The asterisk indicates that control data have significantly (*P*<.05) higher values than the experimental group. CTRL: control data; MPA: moderate physical activity.

As to indices related to levels of physical activity (NPA, LPA, MPA, and VPA), the experimental group, compared to control data, showed significantly lower (−84.3%) moderate activity (MPA, Figure 4) and absent vigorous activity, while lightly longer durations of NPA and light activity (LPA) were not significantly different from control data. Both short-term and long-term ICC of indices related to levels of physical activity evidenced excellent test-retest reliability.

**Figure 5.**
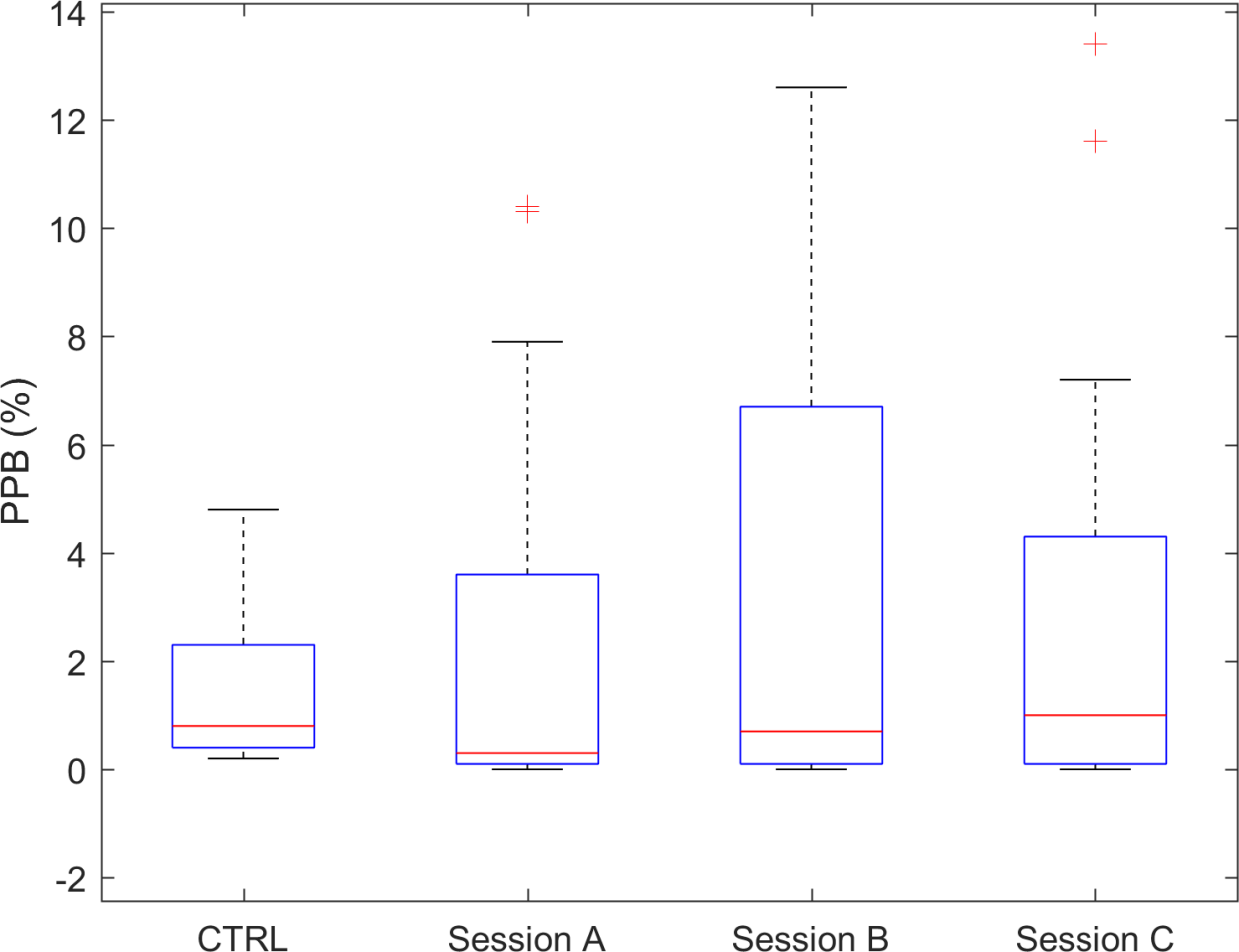
PPB index for CTRL and for the 3 sessions of the experimental group. Red “+” marks indicate outlier individuals. CTRL: control data; PPB: pseudoperiodic behavior.

The PPB index, related to the percentage of time spent in steady walking, did not show a statistically significant difference between the control and experimental group, though in the latter group, the median values of the index were reduced by −37.5%. A perusal of the index values’ distributions (Figure 5) lets us also detect the presence of a large number of cases showing larger values, and sometimes extremely larger values, than normal PPB values, in contrast with the average trend. The latter observation accounts for the so-called “wandering” behavior adopted by a subgroup of cases, who spent most of their time walking along the unit’s corridor. The test-retest reliability of the PPB index was excellent.

**Figure 6.**
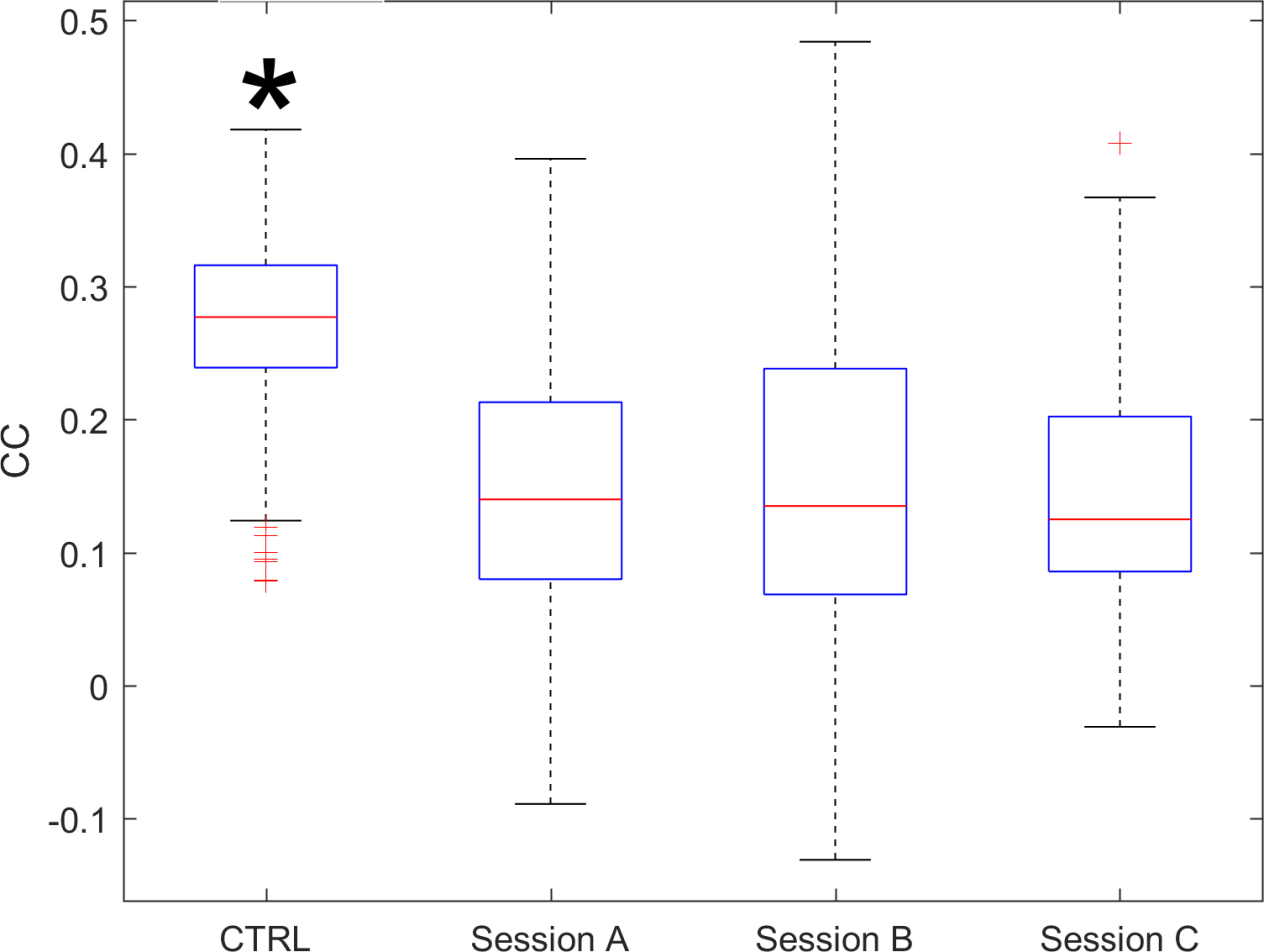
CC index for CTRL and for the 3 sessions of the experimental group. Red “+” marks indicate outlier individuals. The asterisk indicates that control data have significantly (*P*<.05) higher values than the experimental group. CTRL: control data; CC: cross-correlation coefficient.

The CC index, quantifying the accordance between the activity profiles of different participants, is compared with control data obtained from healthy age-matched individuals not living together (retrieved from the study by Rabuffetti et al [11]). The CC values (Figure 6) of the experimental group were significantly smaller, about half of the control values’ (−53.1%), and demonstrated a good test-retest reliability.

**Figure 7.**
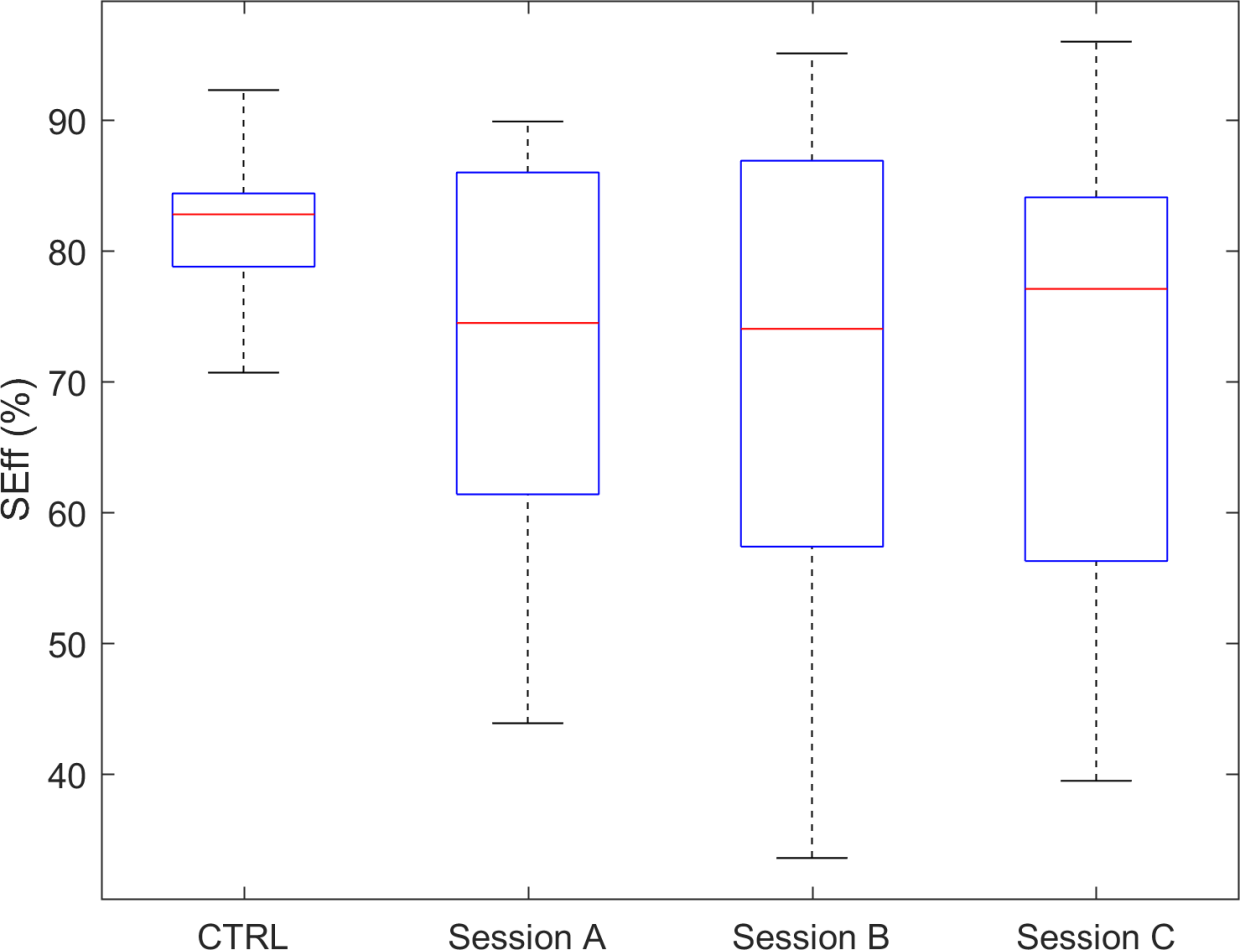
SEff index for CTRL and for the 3 sessions of the experimental group. CTRL: control data; SEff: sleep efficiency.

**Figure 8.**
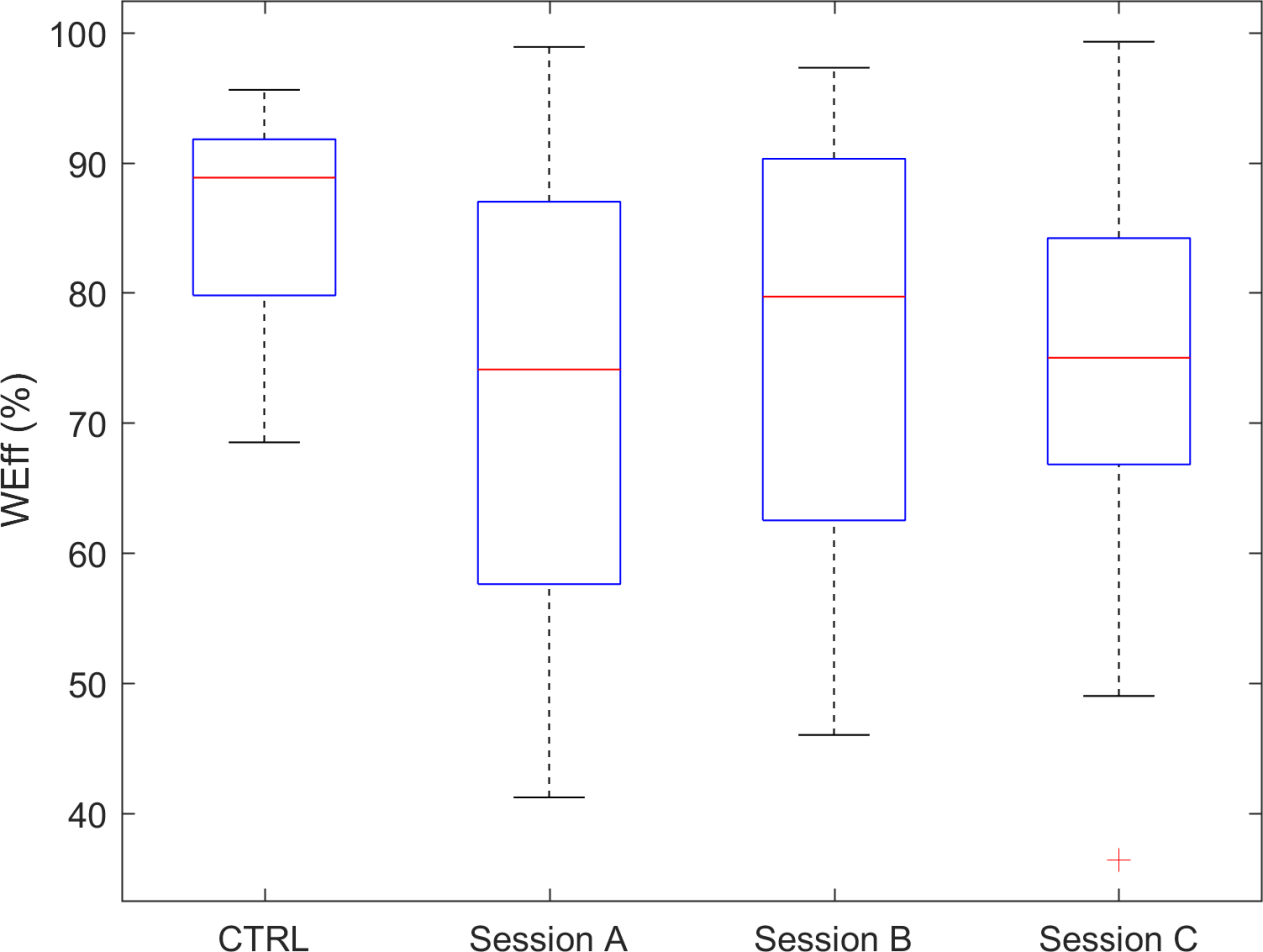
WEff index for CTRL and for the 3 sessions of the experimental group. CTRL: control data; WEff: wake efficiency.

The indices quantifying efficiency of wake time (WEff) and sleep time (SEff), that is, the percentage of the a priori fixed duration of, respectively, daytime and nighttime, showed a tendency toward a smaller efficiency in the experimental group than in controls, which failed to achieve statistical significance after correction for multiple comparisons. Though a perusal of values’ distributions for SEff (Figure 7) and WEff (Figure 8) pointed out the occurrence of a not negligible number of individuals showing fairly reduced efficiency indices’ values. Interestingly, although these 2 indices did not show a between-group significant difference, their test-retest reliability proved to be excellent (ranging from 0.74 to 0.89).

The indices related to location tracking (PRI, PUB, and corridor) could not be compared to control data, which were unavailable due to the specific nature of the measurement. There was no significant intersession difference, and the test-retest reliability was generally fair (it was good just in 1 of 6 ICC values).

These location tracking indices’ boxplots and the between-sessions scatterplot are available as a slide presentation in Multimedia Appendix 1.

## Discussion

### Features of the Proposed Monitoring Methodology

Instrumented methods for physical activity detection and tracking are valuable tools for studying individuals with dementia [42], complementing self-report measures such as questionnaires and surveys.

The presented method has provided a quantification of various aspects concerning physical activities during wake time, sleep, and social interactions among the components of the participants’ group. Besides the characterization of average behaviors as quantified by the various indices, it is worth noting that distinctive individual behaviors, different from the average patterns, were consistently identified as well, as shown also by the high test-retest reliability of the computed indices. In fact, this sensitivity aspect supports the use of such methods not only in group studies but also in clinical settings where accurate individual characterization and management are essential.

The indices considered in this study were intended to depict a multidimensional figure of each measured participant. Along with indices related to levels of physical activity (NPA, LPA, MPA, and VPA) [38], indices concerning sustained walking (PPB), social interaction (CC) [11], quality of nighttime rest (SEff), and daytime vitality (WEff) were included.

The indices’ definitions are generally self-explanatory. Only the rigid identification of wake-time as daytime and sleep-time as nighttime adopted in the wake and SEff indices may be regarded as a critical choice. An alternative choice would be a functional identification of wake epochs and sleep epochs [43]. In fact, the relatively simple original definition of SEff in sleep studies (percentage of time asleep on the total time spent lying in bed, with these 2 measures obtained, respectively, from EEG (electroencephalography) polysomnography and observation log) required a redefinition of the indices when wrist accelerometry was introduced [44]. Interestingly, a literature review [45] has highlighted that the use of wrist accelerometry in sleep studies offers several advantages, such as high participant compliance, but also drawbacks, including the potential under- or overestimation of sleep indices. In this study, a rigid timeline approach was adopted to meet the requirements of clinical partners, who were specifically interested in monitoring residents’ compliance with the daily routines established within the long-term care unit. According to these routines, all guests are expected to be in bed during the “night” interval (11 PM-7 AM) and awake during the “day” interval (9 AM-7 PM). The “day” and “night” time-intervals are separated by 2 transition time intervals, during which “go-to-bed” (7 PM-11 PM) and “get-out-of-bed” (7 AM-9 AM) events may occur. Interestingly, these events do not occur simultaneously across the guests. Consequently, the inclusion of the transition intervals within either the “day” or “night” intervals would have implied the risk of false positive or false negative classifications. Importantly, omitting the transition intervals does not compromise the reliability of the efficiency indices, which are computed over time windows of 8 hours (“night”) and 10 hours (“d”). These durations are consistent with the minimum actigraphic recording time of 8 hours required to obtain reliable indices [46]. Therefore, we consider the rigid timeline approach reasonable and justify the inclusion of the transition intervals as a strategy to improve the reliability of the computed indices.

### Features of the Implemented Experiments

The indices obtained in the experimental group were compared with proper age-matched normative data extracted from already available datasets [11,30]. The only exceptions are for the CC index whose normative data were from a younger group (mean age 72, SD 7.6, range 58-87 years) [11], and for the location tracking indices which were based on a specific local network of BLE antennas and therefore did not allow neither the recording of normative data in the long-term care residential unit, nor the option to set up a duplicate of the residential unit for healthy older adults, was considered.

The installation of the system of BLE antennas was performed in the weeks preceding the experiments without major interference with the daily flow of activities in the care unit. The accuracy of the system (ie, the correct classification of the room where the specific BLE beacon was in) was obtained as a result of a calibration procedure following the installation. The experimental data collection was carried out smoothly without any potentially confounding events, particularly, as described by the clinical logs, the participants constantly resided in the long-term care unit without leaving it. The participants occasionally received nursing assistance in their activities, particularly hygiene-related ones, in which partly passive movements could be interpreted as LPAs. However, these nursing activities were limited in time (few minutes), therefore were not considered to alter the experimental outcome significantly. All accelerometric recordings were a posteriori checked for identifying sensor removal (a constant baseline clearly distinguishes these from actual measurement, even from a still sleeping participant) and eventually tagged to exclude these time intervals from further analyses.

### Discussion on Outcome Indices Compared With Reference Data

The indices related to physical activity (NPA, LPA, MPA, and VPA) evidenced significant differences between the group of persons with dementia and age-matched healthy participants: a slightly larger, though not significantly different after *P* value correction, duration of phases of NPA, a comparable LPA, a significantly reduced duration of MPA, and a substantially absent VPA. This figure is compatible with a commonly expected behavior in a nursing ward: increased sleep or rest, even in a seated position, particularly during the day [47], and drastically reduced activities requiring a relevant physical involvement [48]. A perusal of the individual data allowed us to identify individuals who showed activity profiles that differ from average behavior: particularly, 2 cases evidenced a very low value of NPA, which was compensated in 1 case (S18) by large values of LPA, while the other case (S23) evidenced the largest values of MPA. Interestingly, S18 had the lowest Barthel score among participants, while S23 was the youngest and had the lowest Tinetti score.

The index PPB, related to a pseudoperiodic motor behavior, is to be interpreted as an index of steady walking. Indeed, while the PPB index was, on average, not significantly different between controls and this study’s group, a small subgroup of participants showed higher than normal PPB (S25, S23, and S07 above all controls in all 3 sessions, S13, S06, and S09 above at least 9 of 10 controls in all 3 sessions). The motor behavior of these participants resembles the so-called “wandering” behavior [49,50], a continuous, uninterrupted walking along the allowed paths, such as up and down a main corridor. Specifically, in this study’s group, 6 individuals had this wandering behavior, clearly detectable in PPB scatterplots. Interestingly, this behavior is very consistent and can be observed also in repeated sessions, weeks, or months apart.

The CC index, quantifying the accordance between MA profiles of any possible couple of participants, is the key variable in the so-called social actigraphy approach [11] that aims at identifying congruent synchronized behaviors induced by the social proximity (in the original paper due to the marriage or cohabitation, in this study due to the common belonging to the community living in the long-term residential unit). In the presented data, the CC index showed significantly lower values in the experimental group, compared to reference data published in literature [11]. This result was initially unexpected, since a simple assumption implies that a tight social community, such as the one composed of guests of a nursing home, should be characterized by higher between-subjects CC values. The presented data evidenced, on the contrary, that participants with dementia show larger between-subjects differences in the MA profiles (corresponding to lower CC values) than observed in healthy participants. This outcome is surprising when the attention is all upon the social context, with common activities such as having meals together or participating in socialization events, since it would imply a certain “temporal synchronization” of motor behaviors. On the contrary, this result could be explained by the fact that people with dementia are known to often show apathy and withdraw from social interactions [51], and sometimes individuals with dementia may even show sociopathic traits [52].

The index SEff showed, on average, comparable values between healthy participants and persons with dementia. Interestingly, a large number of this study’s group was characterized by lower-than-normal values, thus evidencing a troubled management of the hours eligible for sleep or at least rest. The repeatability of these lower-than-normal values lets us identify a subgroup of the guests of the residential ward that need special attention concerning the management of the nighttime. Without entering into specific details, several actions can be performed, including a revision of pharmacological or nonpharmacological interventions [53-55]. Nonetheless, the present method lets us consider also more practical choices such as a change of the bedroom and related roommate. The index WEff evidenced a tendency, not statistically significant after *P* value correction, of lower-than-normal values for people with dementia, implying reduced percentage of activity time and increased resting, possibly daytime napping [56]. Symmetrically with previous comments on SEff, it is possible to identify a small subgroup that evidenced a higher-than-normal WEff, thus presenting themselves as the guests who never sleep during the day and are always active.

The indices related to location tracking (PRI, PUB, and corridor), that is, the percentages of total time spent in specific indoor locations, have no reference normative data. The interpretation of the observed values relies on comparison with other indices. The PRI values, representing the time spent in PRIs, were found to be slightly larger than NPA, the time characterized by no activity. This result is consistent with the fact that PRIs were mostly used for resting and sleeping. Conversely, the sum of PUB and corridor durations (ie, the time spent in any public space) was found to be slightly lower than the sum of LPA, MPA, and VPA durations (ie, the time spent at any level of activity), thus implying that MA might occur even in PRIs.

### Test-Retest Reliability of Outcome Indices

The ICC values of the physical activity indices (NPA, LPA, and MPA) were excellent either in the short-term or in the long-term, with just a slight decrease of the values in the latter case. No ICC was computed for the VPA because vigorous activity was substantially absent in all cases. When considering PPB as a proxy for the amount of performed steady gait, the ICC was excellent in both short-term and long-term, and, to our surprise, it scored a higher value in the long-term. We are not willing to interpret this latter point as significant of some underlying phenomenon; rather, we consider it the result of a stochastic effect. Lower, but still good, ICC values were observed for the social actigraphy index CC. In fact, the CC values were substantially unchanged in all sessions, both after 3 weeks and after 3 months. As the CC values depicted a figure of a substantial absence of relations among the MA profiles of participants, even compared with control data, our interpretation is that people with dementia living in long-term care residences show signs of social withdrawal in their lifestyles as well as peculiar behaviors that may occur, such as the wandering one that largely differs from the inactive one which characterizes the majority of guests. Excellent ICC values were observed for SEff in the short-term. Indeed, participants tended to keep their night habits, which included either high SEff behaviors or persons who spent a large part of the night asleep. It is to be noted that the clinical management rules of the experimental residential unit excluded a general use of antipsychotics [57], thus increasing the possibilities for a person to be free to stay awake and even wander, as it was seldom observed, during the night. The test-retest reliability of WEff was between good and excellent. As for night habits, even during the daytime, participants had different behaviors (some stayed awake the whole day and others had frequent day naps). Those behaviors were individually consistent and repeatable. The location tracking variables, that is, the percentage time spent in different locations inside the residence unit, showed mainly fair values of reliability. The relatively lower values of the location tracking indices’ ICC disagreed with the higher ICC values of the accelerometer-based indices. It is an open question how much of this low reliability of the location tracking indices is due to the actual, and different from the acceleration, nature of measured variables, and how much is due to a different behavioral performance. As, occasionally, we observed that the system delayed the detection of a displacement of a participant, that is, of the Bluetooth tag he wore, from one space to another one, we believe that these technical occurrences might have decreased the overall reliability of the Bluetooth measure, leading therefore to a decrease in the reliability of the variables which were derived from that system.

The obtained ICC values should be compared to ICC values found in the literature. When considering indices obtained by a questionnaire, a published study reported ICC ranging from 0.53 to 0.68 for total sedentary time, while for more focused domain-specific sedentary times, ICC ranged from 0.36 to 0.76 [24]. Another study concerning a self-administered questionnaire quantifying physical activity indices reported ICC values ranging from 0.56 to 0.91 for physical activity indices [25]. A questionnaire administered to caregivers of persons with dementia scored an ICC ranging from 0.87 to 0.95 [26]. When considering sensor-based assessments, a study on patients with knee osteoarthritis reported ICC ranging from 0.70 to 0.93 for physical activity indices [29]. In a gait monitoring approach in older adult participants, it was observed that higher repeatability was observed among the prefrail or frail group (ICC>0.78) compared to nonfrail individuals (0.39<ICC<0.55) [28]. In a study, in which the accelerometer data were processed with different algorithms, the test-retest reliability of the physical activity indices was generally very good to excellent (ICC=0.70‐0.90) [27]. All considered, the test-retest reliability found in the present study is comparable with the already published literature.

### Limitations

The presented experiment has some limitations. First, the location tracking method required the installation of a hardware infrastructure embedded in the building, which is not movable to another setting (this limitation obviously does not apply to the wrist accelerometer setup). As a consequence, control data obviously cannot be obtained from healthy age-matched individuals living in the same environment. This limitation might be overcome in the future, in the light of a rapidly evolving technological advancement, if detection antennas for an ultrawide band become ubiquitous indoors [58] or with AI-enabled surveillance cameras, provided that privacy concerns are solved [59]. Second, the activity tracking method had a compliance which was not 100% because some individuals did not agree to wear the wrist sensors or removed them during the experiment, resulting in a generic discomfort, though the clinical partners had preliminarily warned about these refusals as a consequence of the dementia affecting the unit residents. In the latter cases, when the wrist sensors measured for more than 24 hours, the data were retained for multiples of 24-hour durations, and outcome variables were computed on the available full days. Nonetheless, such occurrences were seldom observed. Despite the authorization given by the legal tutors, our participants, persons with dementia, were still able to refuse to participate or at least to reject the wrist or ankle sensors because they felt disturbed by their presence. The dimension and location of the sensors might play a role in their refusal, so it may be expected that future technological developments, letting smaller sensors be embedded in the garments or fixed into adhesive patches, will reduce this noncompliance. Interestingly, compliance problems arose mostly for the wrist sensor, as the participant could perceive and grasp it. Third, the control group for the accelerometric data was built from an available dataset of healthy older adults living in the community [11,30] to be comparable as to average age, but the age range was narrower (80‐87 years), 9 of 25 participants were above the maximum control age and 7 of 25 participants were below the minimum control age. This is due to the difficulty in recruiting healthy older adults in their nineties living in the community [60] while younger healthy older adults were excluded in order to have a matched average age. Fourth, the choice of monitoring activities with wrist accelerometry (adopted in 22% of the published actigraphic studies) was critically compared with the option of performing waist accelerometry (in 48% of the studies) [61], as an accelerometer on the waist is closer to the body center of mass and, therefore, considered more informative about physically engaging activities. The wrist choice was justified by the relevance of upper limb movements in older adult residents in a long-term care unit, as locomotor activities might be quite reduced, and by feasibility reasons, as an accelerometer positioned on the lower back with a belt would greatly interfere with autonomous bathroom activities of older adults, would not have had a stable position, and would be disturbing when lying in the bed. Moreover, the integration of a gyroscope sensor into the monitoring watch was not useful, and therefore dropped, as gyroscopes are mainly involved in activity classification, which was not an objective of this study.

### Conclusions

In conclusion, the method involving activity tracking by a wearable accelerometer and location tracking through the detection of a wearable Bluetooth beacon by a network of Bluetooth antennas was proven to be feasible. The computed indices quantified the activities and social behaviors of the long-term care unit guests, providing a novel multidimensional characterization of the individual guest behavior, which considers the physical activities, the WEff or SEff, as well as the social interactions in terms of agreement between the activity profiles among participants. Though the results about activity levels in persons with dementia are in line with intuitive knowledge (ie, people with dementia move less than age-matched healthy participants), the assessment provided novel evidence against intuitive knowledge when quantifying the strict and continuous social interactions involved in a residential long-term unit. Such interactions, quantified by the CC index, resulted in lower values than those observed among healthy older adult couples not even living together. Apart from behavioral evidence, the fundamental novel information provided by this study consists of the quantification of the test-retest reliability of the computed indices, which resulted in good to excellent values, thus possibly supporting future power analyses in designing longitudinal studies on the activity tracking and localization of persons affected by health problems or frailty, and particularly by dementia or cognitive decline. These test-retest features complement the ones already available in the literature concerning studies on healthy adults [27], on walking bouts in frail older adults [28], and in people with orthopedic problems [29]. The results presented here will possibly support future studies able to characterize the effects of aging and changing social situations with a longitudinal design, or the effect of a possible therapy in a randomized controlled trial design. Interestingly, the methodology proved to be able to clearly identify behaviors, such as, for example, the wandering behavior, expressed by a minority of individuals inside this study’s group of persons with dementia, thus supporting a possible use of the methodology in clinical settings, either as a way to characterize the individual or a means to detect changes in their behavior. Moreover, the computed indices might support a clinical team in improving the guests’ experience [62] and in tailoring a personalized and coordinated across-subject rehabilitation program.

## Supplementary material

10.2196/70188 Multimedia Appendix 1 Boxplots and scatterplots are not included in the main text.

## Abbreviations

BLE: Bluetooth Low Energy
CC: cross-correlation coefficient
EEG: electroencephalography
ETS: Ente del Terzo Settore
ICC: intraclass correlation coefficient
IRCCS: Istituto di Ricovero e Cura a Carattere Scientifico
LPA: light physical activity
MA: motor activity
MET: Metabolic Equivalent of Task
milli-g: one-thousandth of an unit gravity acceleration (1 g about equal to 9.81 m/s^2^
MPA: moderate physical activity
n.adm.: not administered (said of a clinical test)
NPA: no physical activity
PPB: pseudo periodic behavior
PRI: private room
PUB: public room
SEff: sleep efficiency
VPA: vigorous physical activity
WEff: wake efficiency
