# Supplementary material for: Definition and Test-Retest Reliability of a Monitoring Method Integrating Accelerometric Actigraphy and Bluetooth Indoor Location Tracking Applied in a Long-Term Residential Unit for Persons With Dementia: Longitudinal Observational Study
Source: JMIR Mhealth Uhealth. 2026 May 21;14:e70188. doi: 10.2196/70188 (PMC13193664; doi:10.2196/70188)
Supplement: Multimedia Appendix 1 — Boxplots and scatterplots are not included in the main text. [file mhealth-v14-e70188-s001.pptx]

## Slide 1
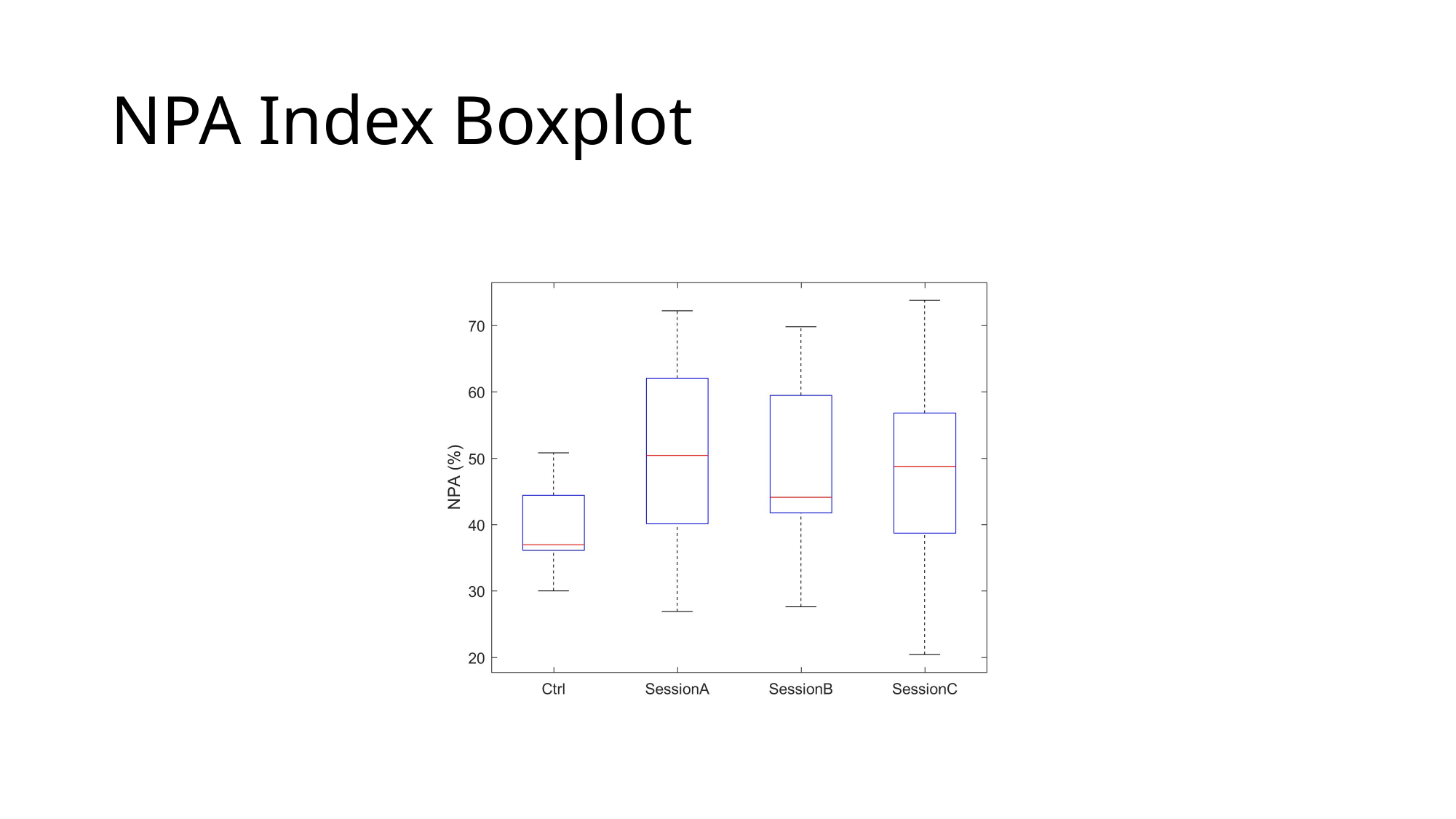

# NPA Index Boxplot

## Slide 2
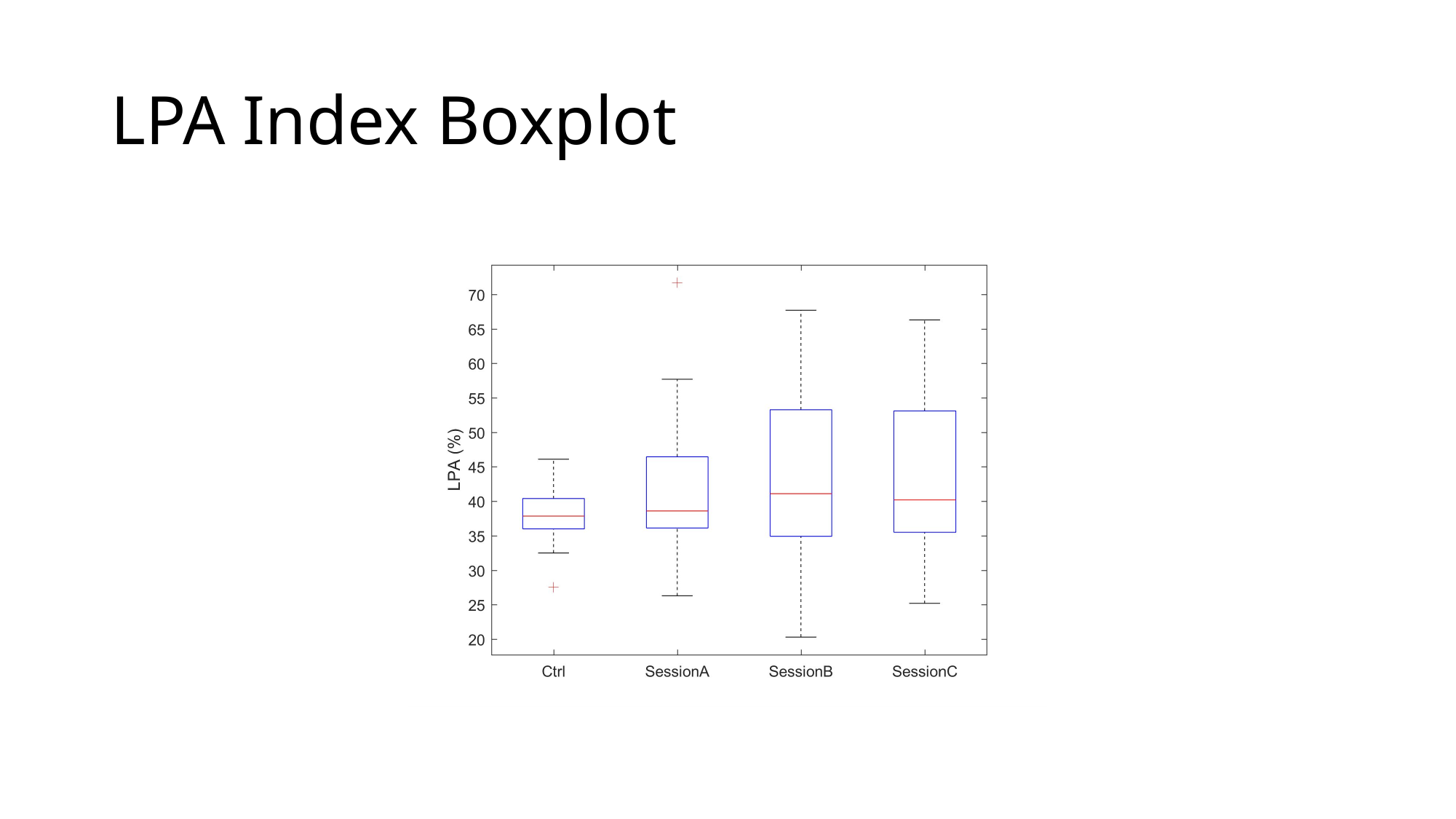

# LPA Index Boxplot

## Slide 3
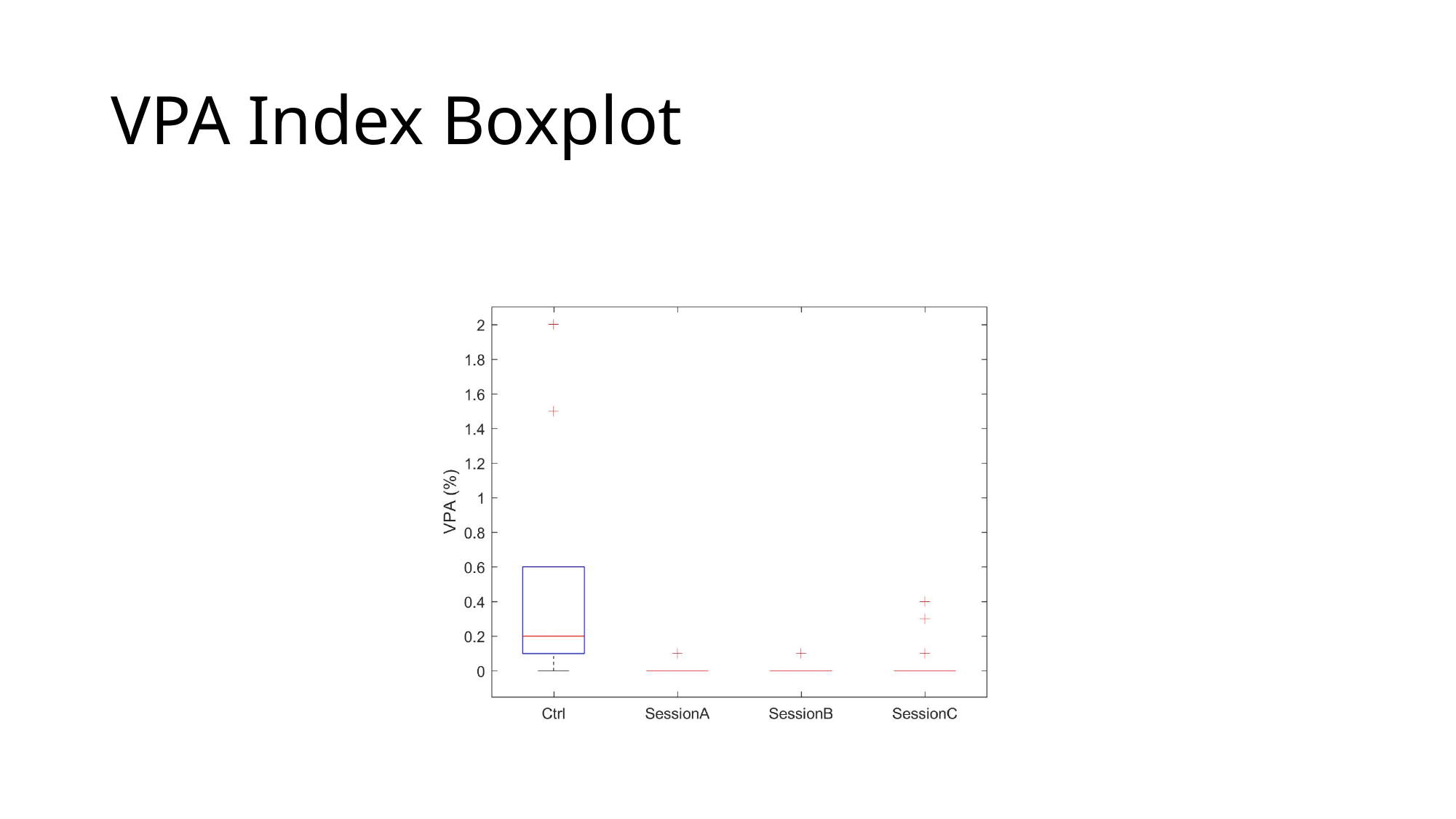

# VPA Index Boxplot

## Slide 4
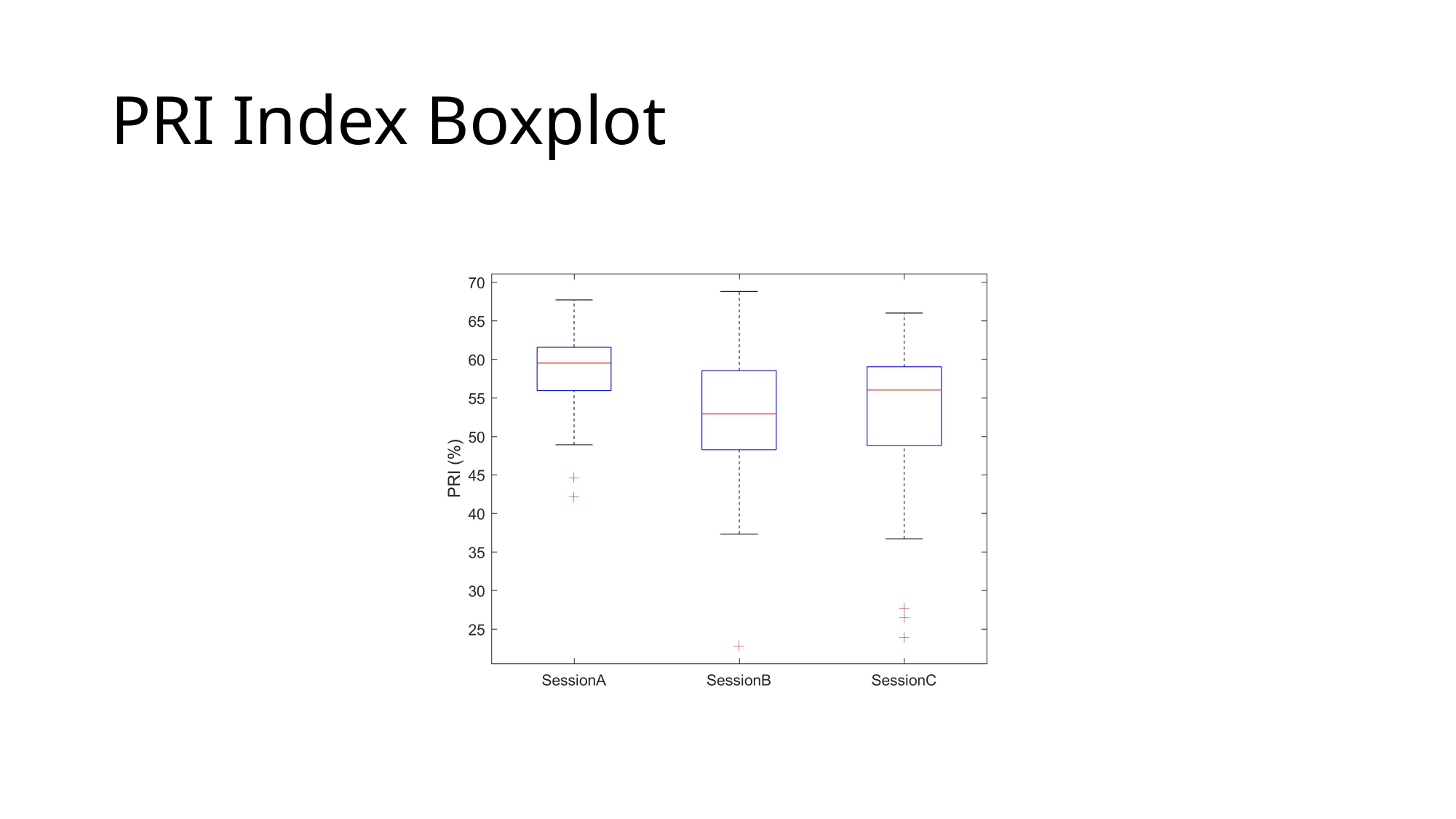

# PRI Index Boxplot

## Slide 5
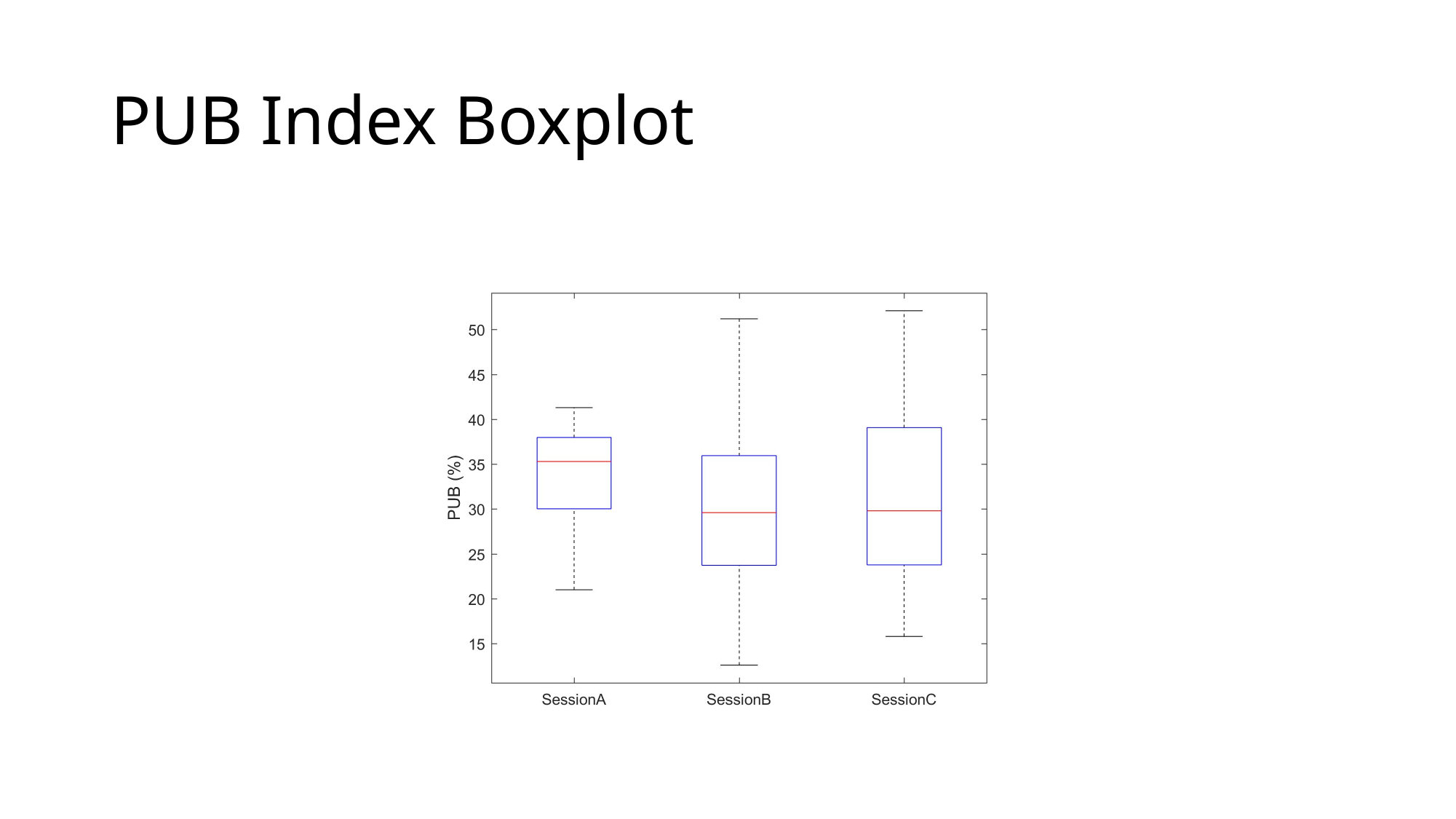

# PUB Index Boxplot

## Slide 6
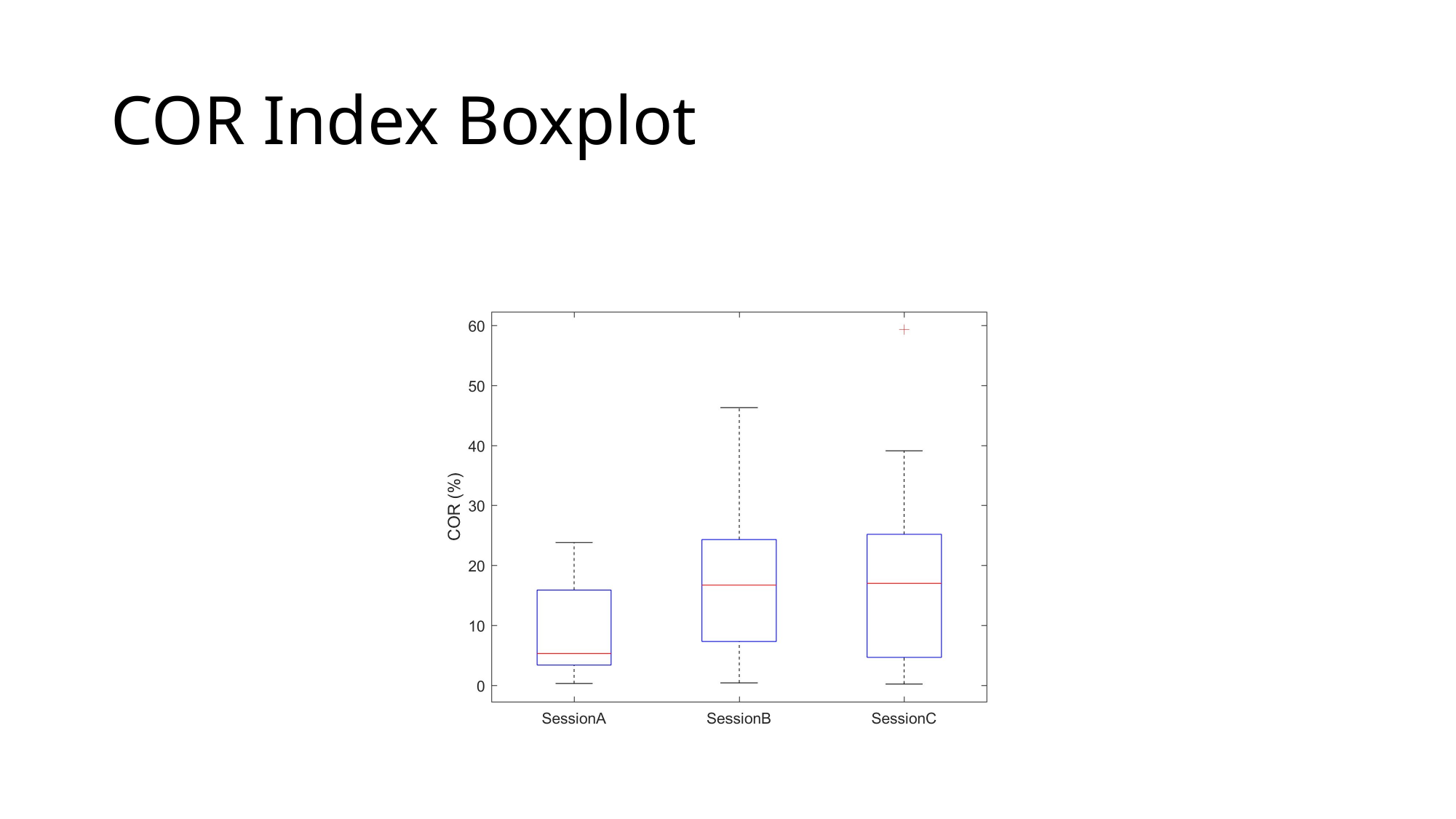

# COR Index Boxplot

## Slide 7
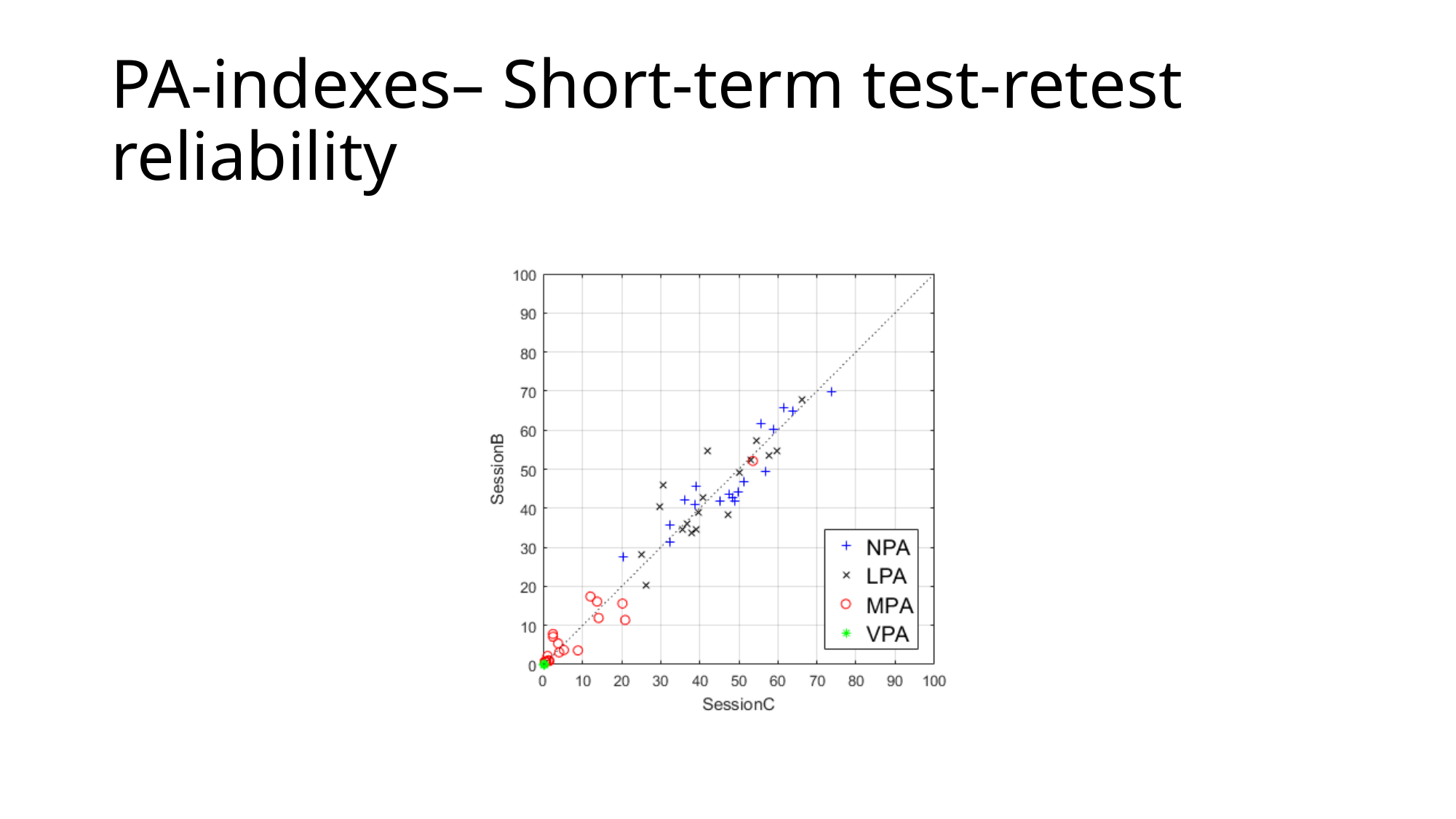

# PA-indexes– Short-term test-retest reliability

## Slide 8
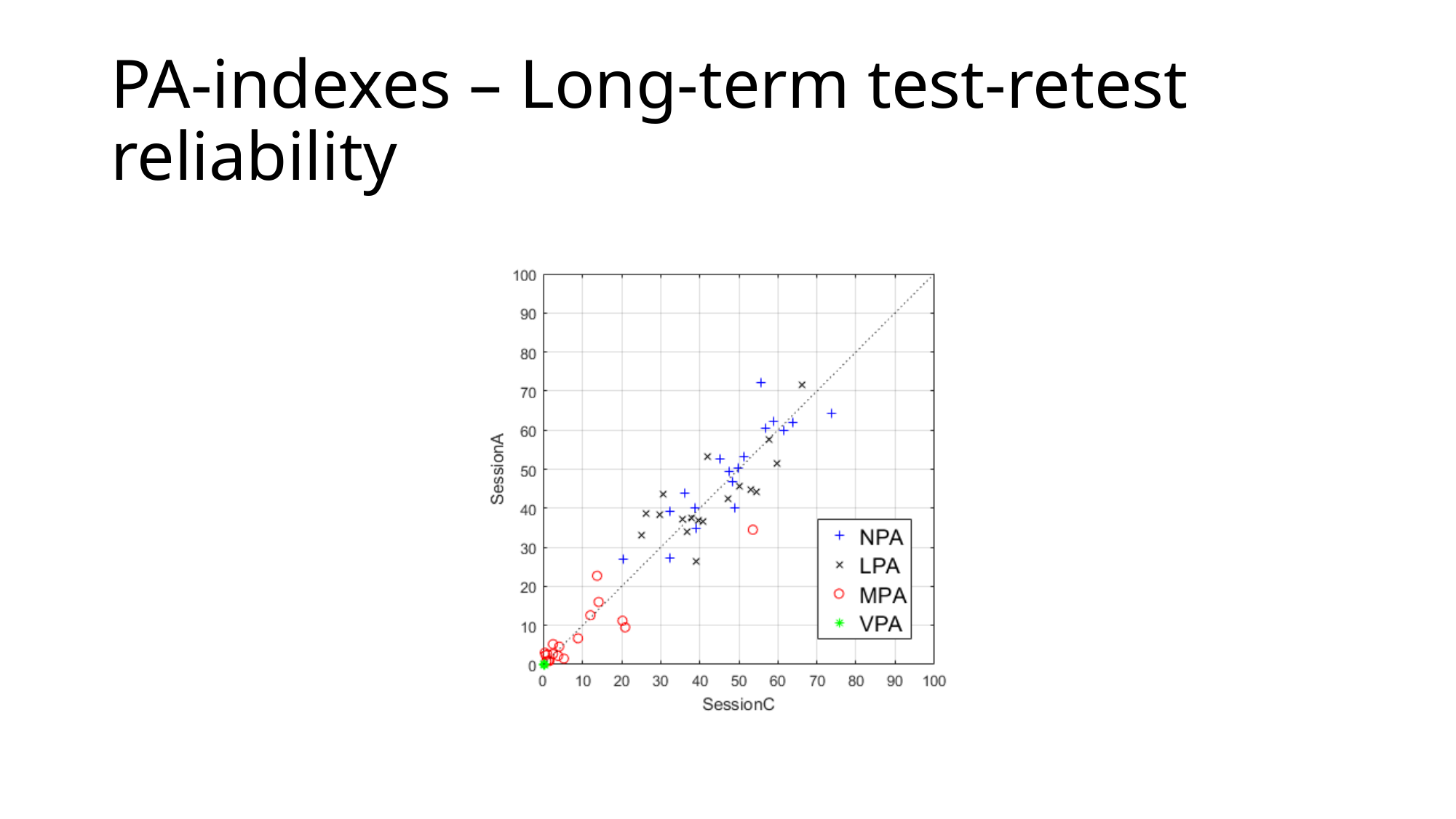

# PA-indexes – Long-term test-retest reliability

## Slide 9
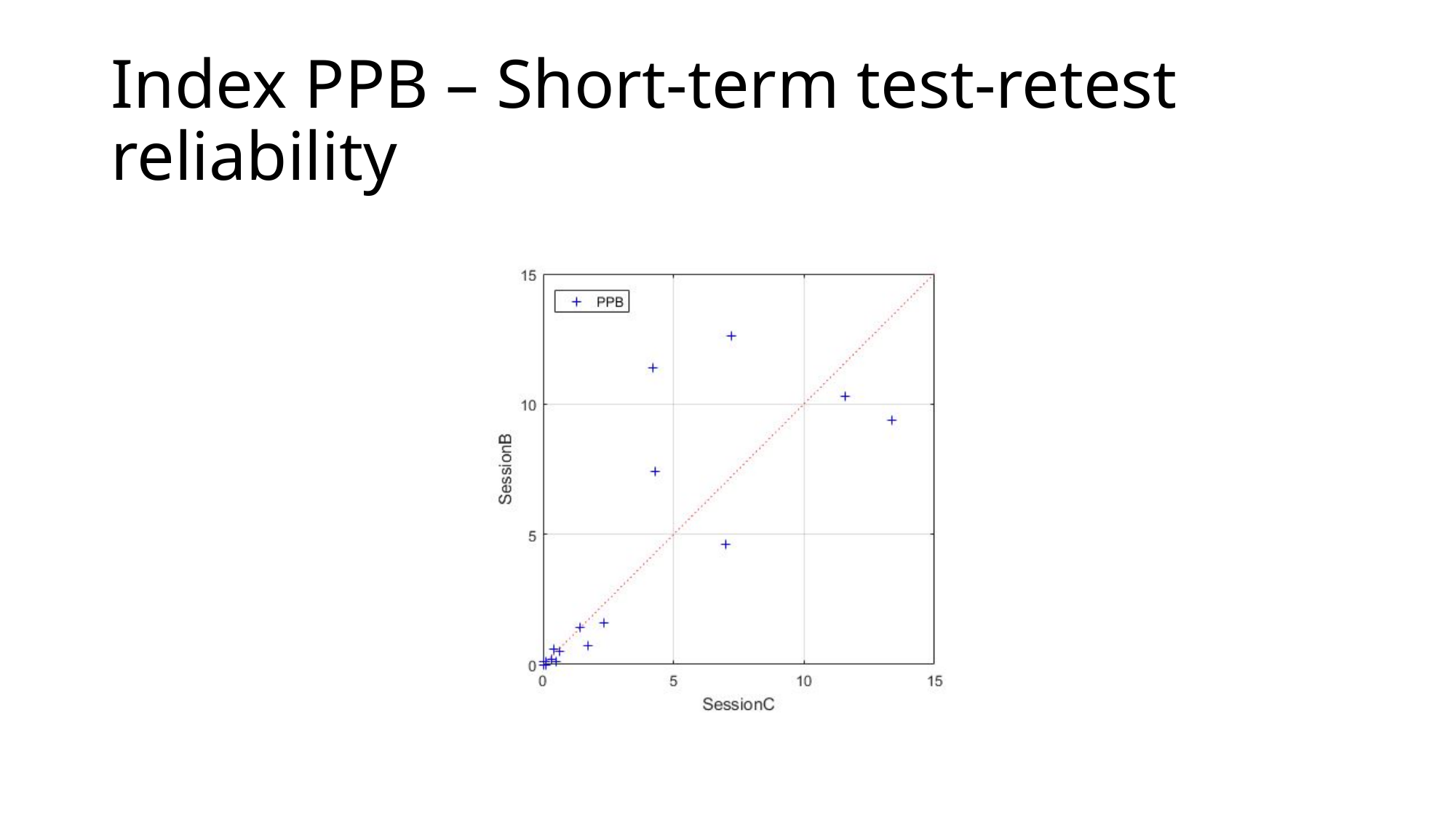

# Index PPB – Short-term test-retest reliability

## Slide 10
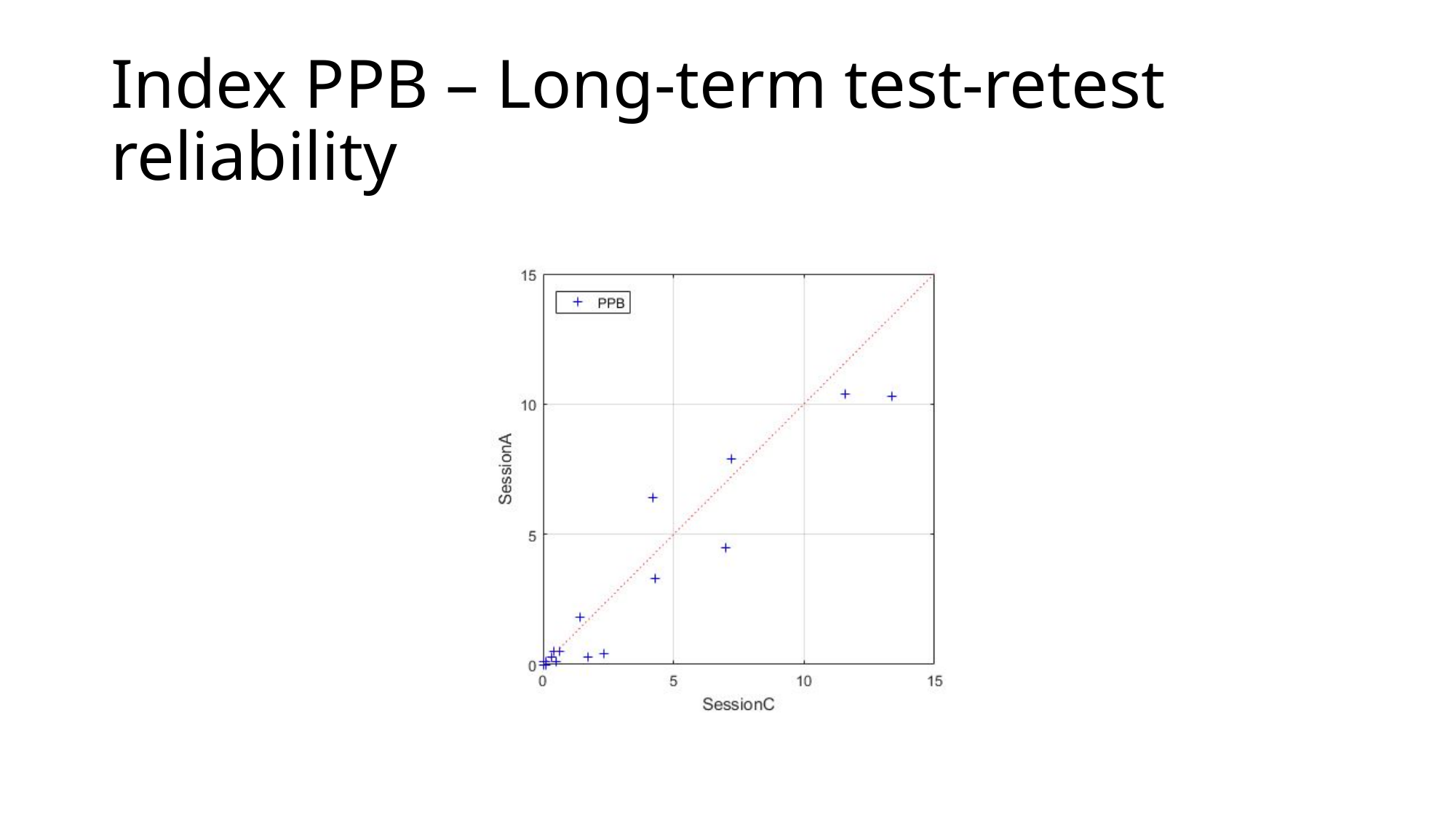

# Index PPB – Long-term test-retest reliability

## Slide 11
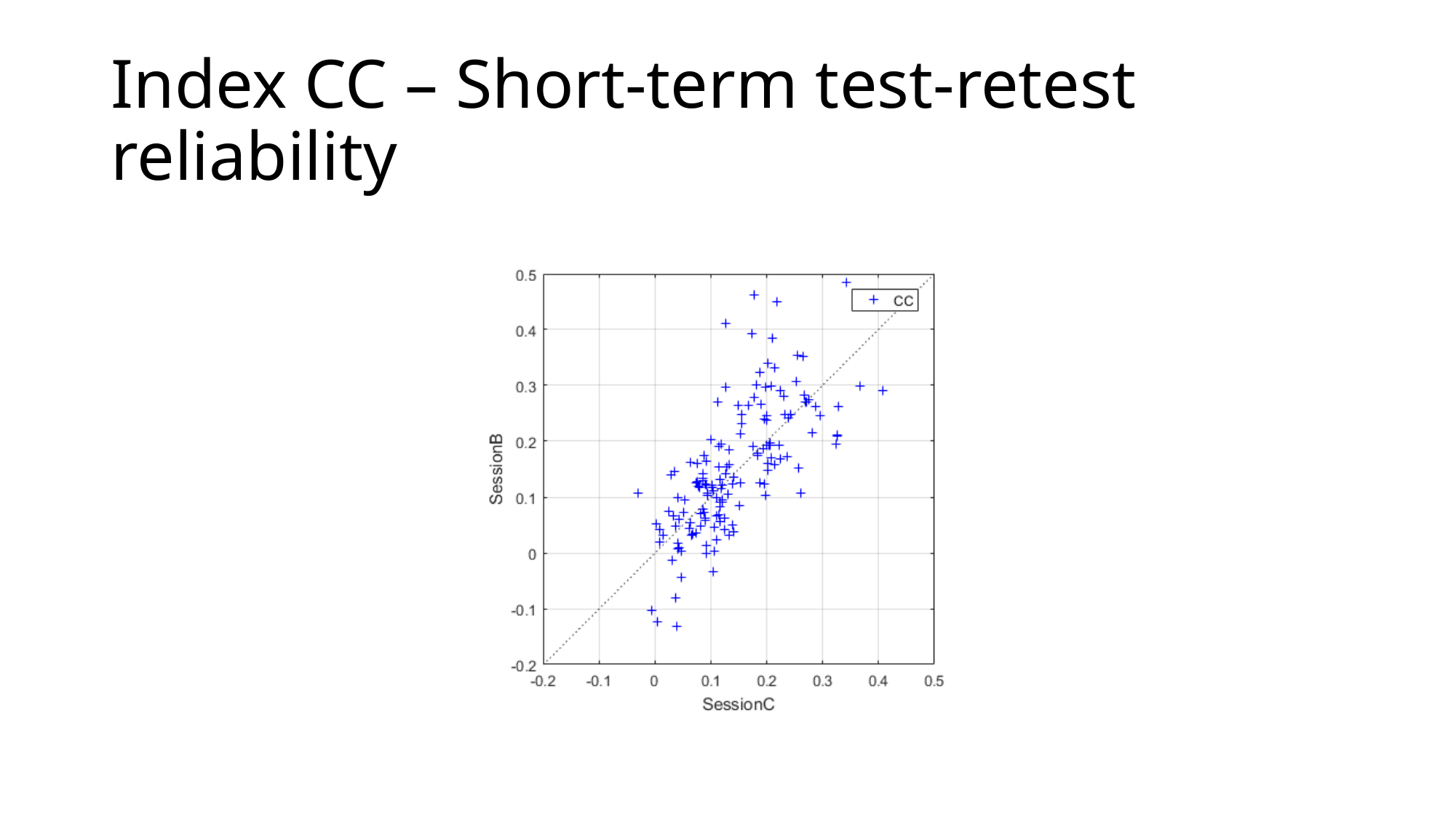

# Index CC – Short-term test-retest reliability

## Slide 12
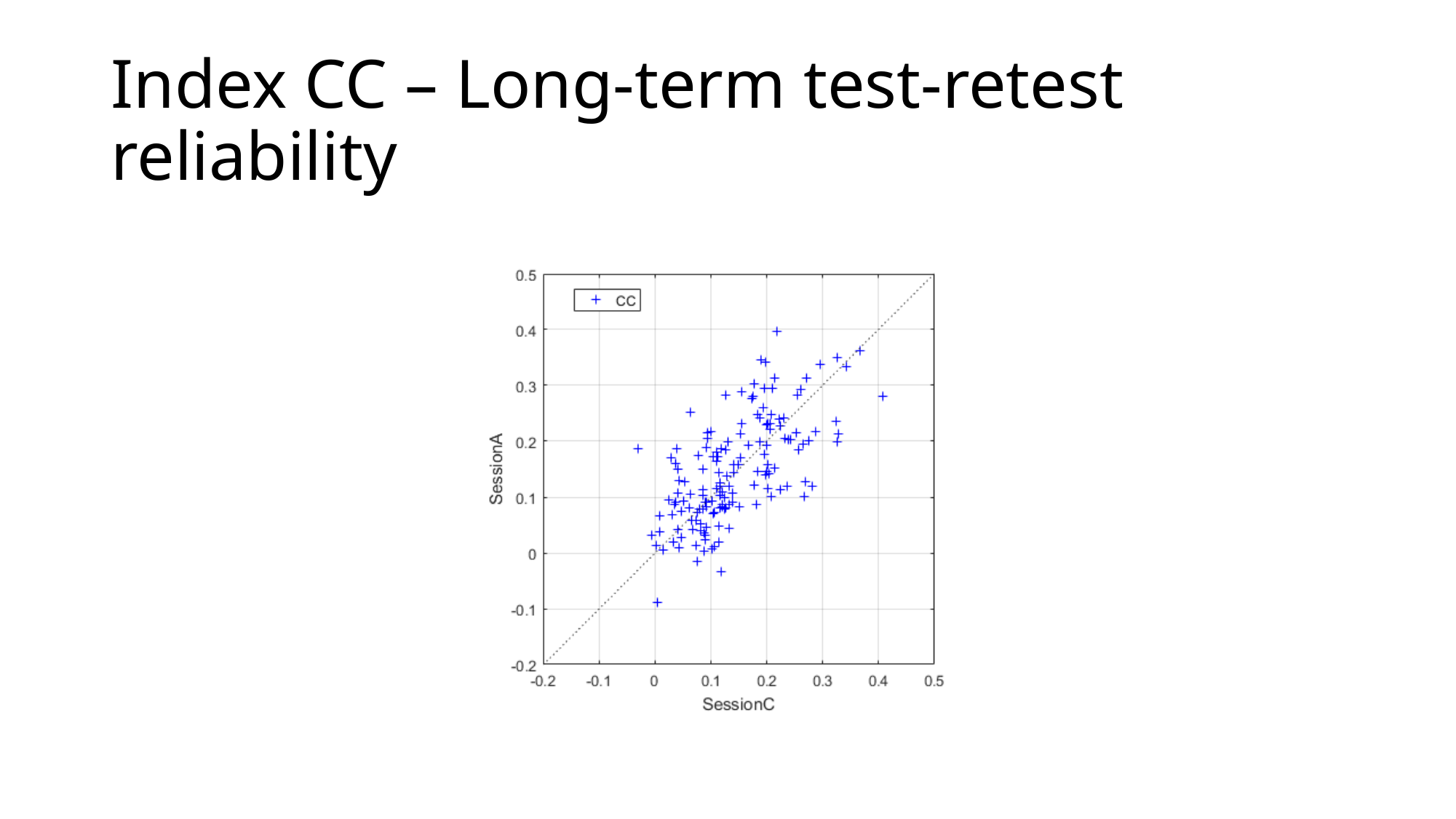

# Index CC – Long-term test-retest reliability

## Slide 13
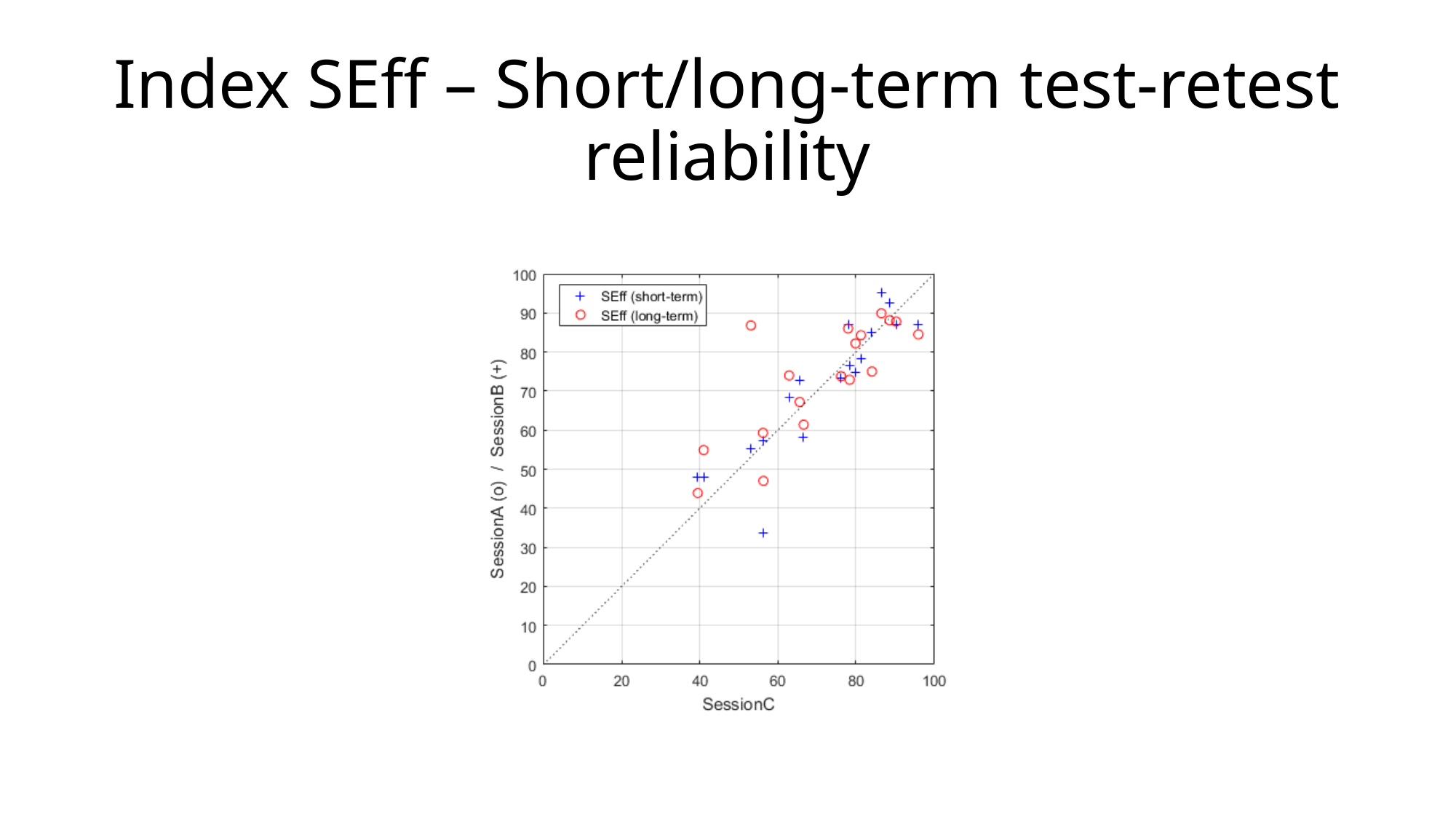

# Index SEff – Short/long-term test-retest reliability

## Slide 14
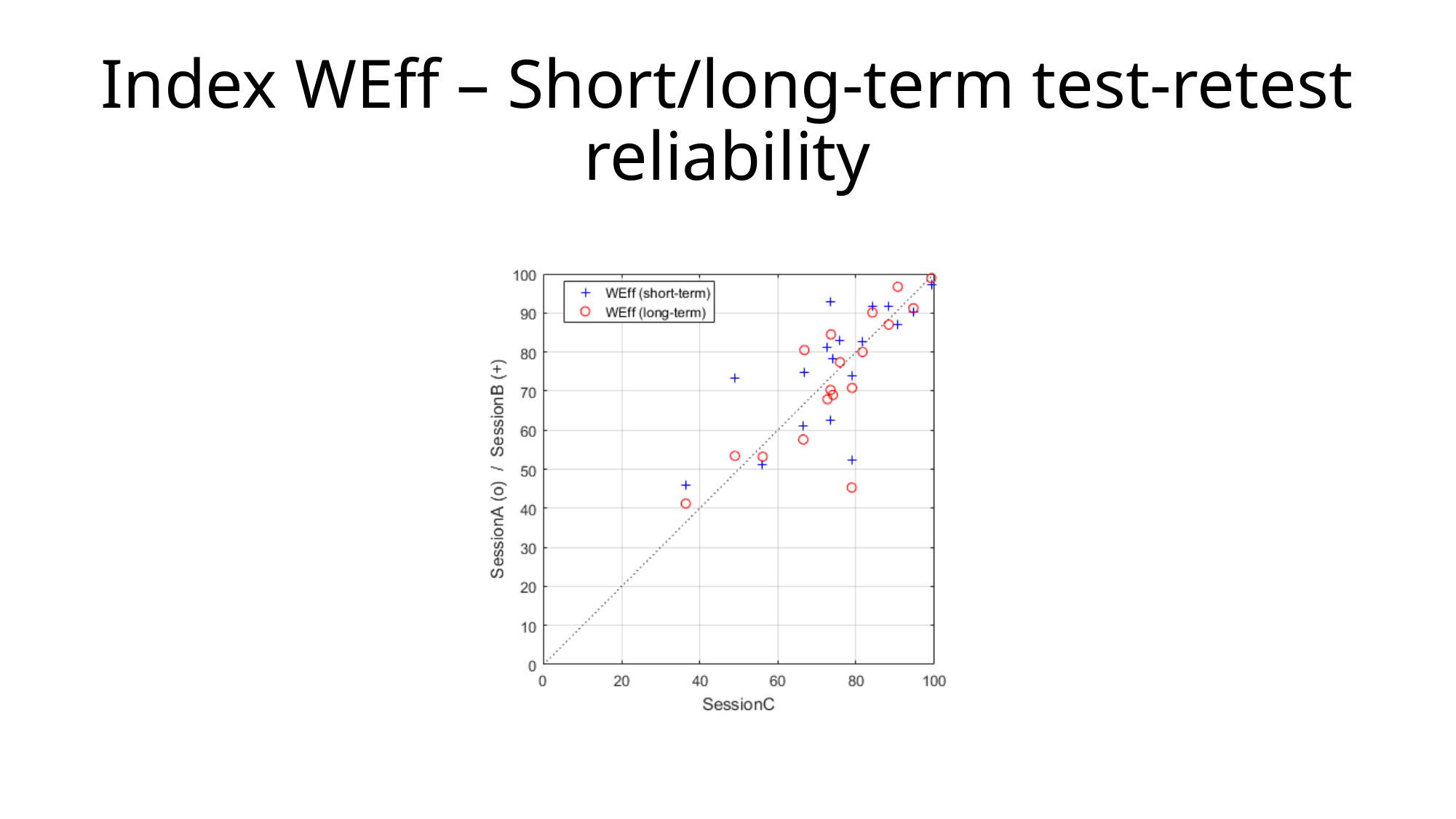

# Index WEff – Short/long-term test-retest reliability

## Slide 15
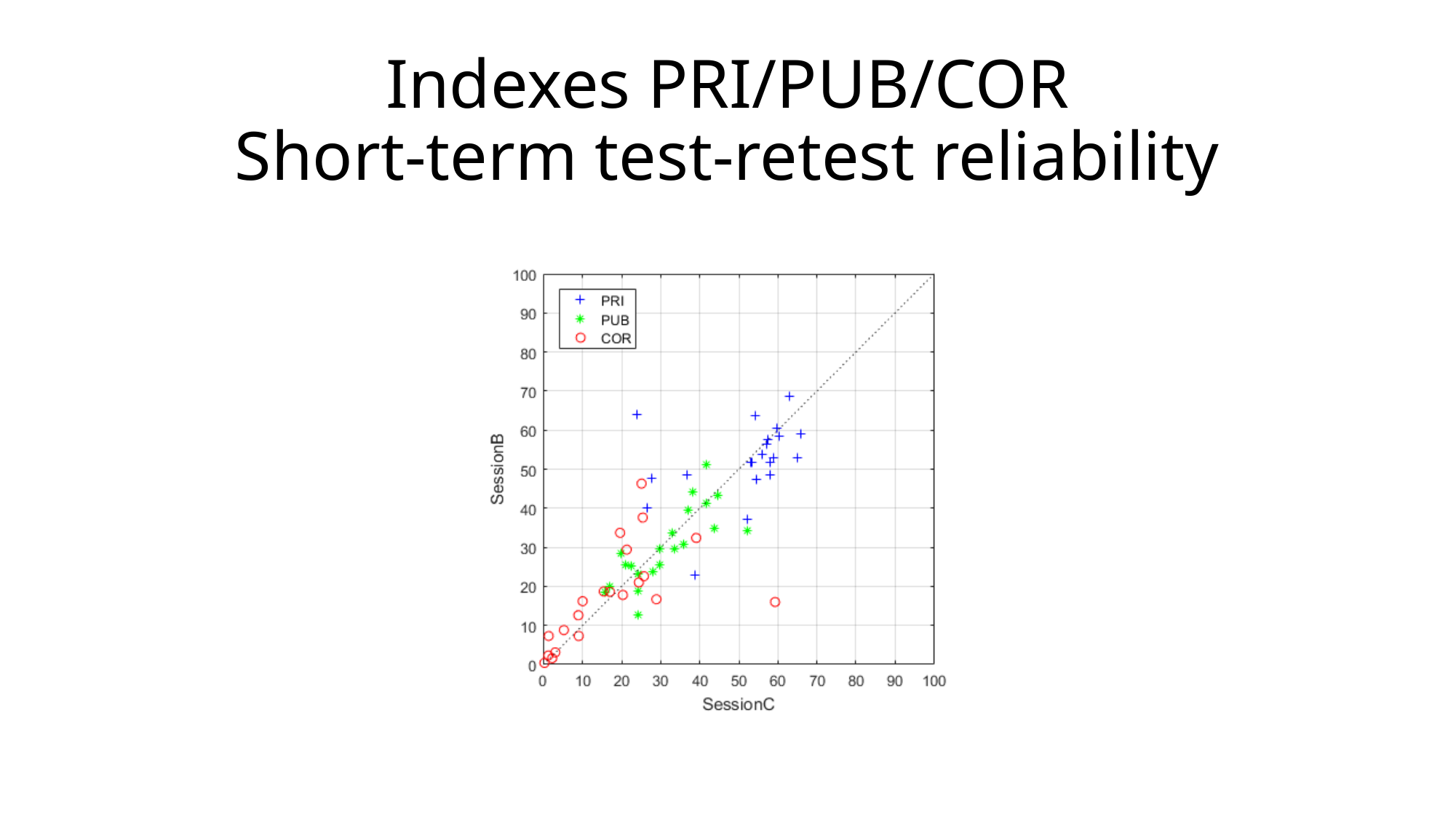

# Indexes PRI/PUB/COR Short-term test-retest reliability

## Slide 16
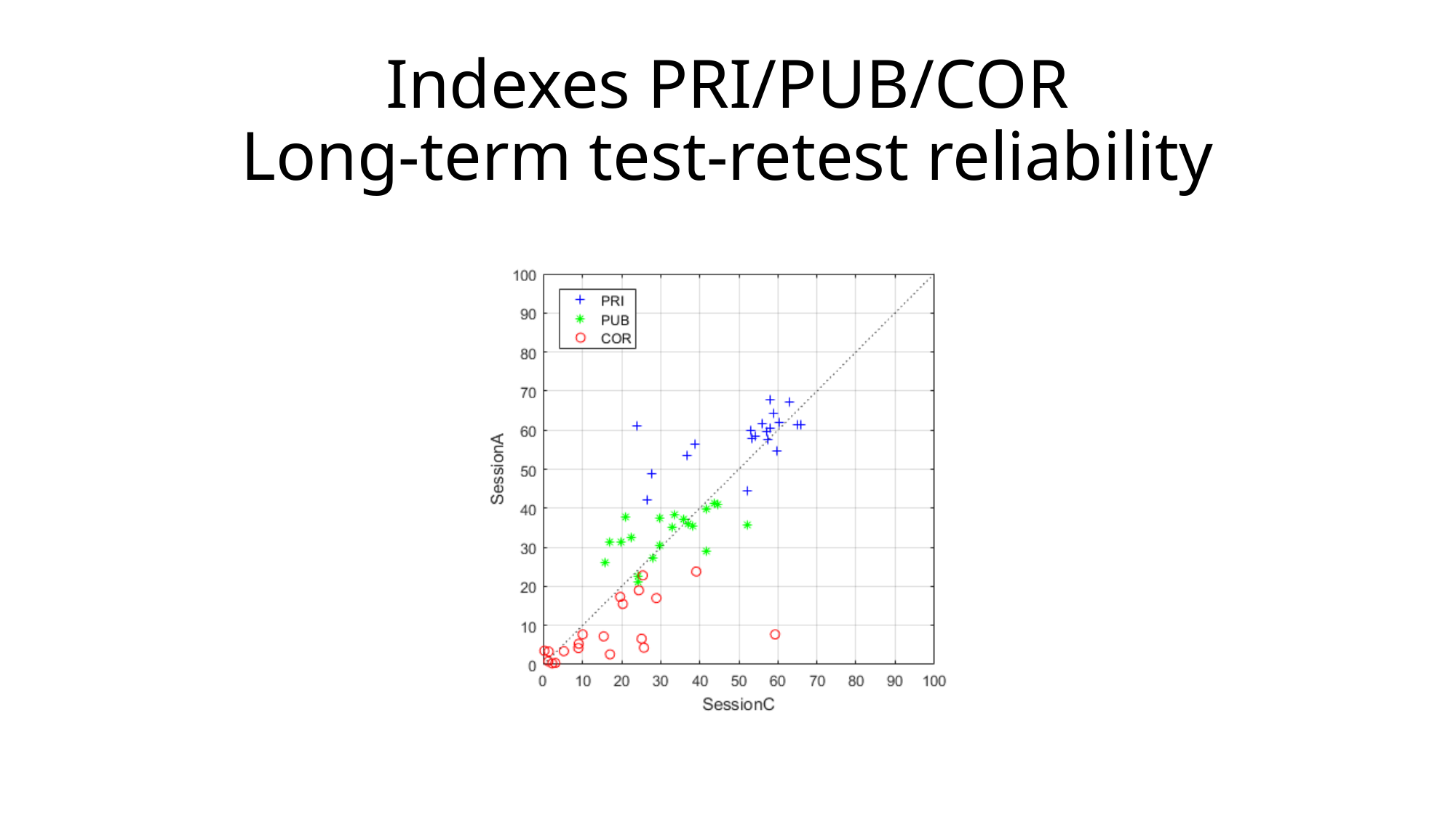

# Indexes PRI/PUB/COR Long-term test-retest reliability
